# Soluble markers of B cell activation suggest a role of B cells in the pathogenesis of systemic sclerosis-associated pulmonary arterial hypertension

**DOI:** 10.3389/fimmu.2022.954007

**Published:** 2022-07-29

**Authors:** Sébastien Sanges, Thomas Guerrier, Alain Duhamel, Lucile Guilbert, Carine Hauspie, Alexis Largy, Maïté Balden, Céline Podevin, Guillaume Lefèvre, Manel Jendoubi, Silvia Speca, Éric Hachulla, Vincent Sobanski, Sylvain Dubucquoi, David Launay

**Affiliations:** ^1^Univ. Lille, U1286 – INFINITE – Institute for Translational Research in Inflammation, Lille, France; ^2^INSERM, Lille, France; ^3^CHU Lille, Département de Médecine Interne et Immunologie Clinique, Lille, France; ^4^Centre National de Référence Maladies Auto-immunes Systémiques Rares du Nord et Nord-Ouest de France (CeRAINO), Lille, France; ^5^Health Care Provider of the European Reference Network on Rare Connective Tissue and Musculoskeletal Diseases Network (ReCONNET), Lille, France; ^6^CHU Lille, Institut d’Immunologie, Lille, France; ^7^Univ. Lille, CHU Lille, ULR2694 – METRICS: Évaluation des technologies de santé et des pratiques médicales, Lille, France

**Keywords:** systemic sclerosis, pulmonary arterial hypertension, B cell, humoral immunity, angiogenesis, pro-angiogenic factors, soluble markers, BAFF

## Abstract

**Introduction:**

Soluble markers of B cell activation are interesting diagnostic and prognostic tools in autoimmune diseases. Data in systemic sclerosis (SSc) are scarce and few studies focused on their association with disease characteristics.

**Methods:**

1. Serum levels of 14 B cell biomarkers (β2-microglobulin, rheumatoid factor (RF), immunoglobulins (Ig) G, IgA, IgM, BAFF, APRIL, soluble (s)TACI, sBCMA sCD21, sCD23, sCD25, sCD27, CXCL13) were measured in SSc patients and healthy controls (HC). 2. Associations between these biomarkers and SSc characteristics were assessed. 3. The pathophysiological relevance of identified associations was explored by studying protein production in B cell culture supernatant.

**Results:**

In a discovery panel of 80 SSc patients encompassing the broad spectrum of disease manifestations, we observed a higher frequency of RF positivity, and increased levels of β2-microglobulin, IgG and CXCL13 compared with HC. We found significant associations between several biomarkers and SSc characteristics related to disease phenotype, activity and severity. Especially, serum IgG levels were associated with pulmonary hypertension (PH); β2-microglobulin with Nt-pro-BNP and DLCO; and BAFF with peak tricuspid regurgitation velocity (TRV). In a validation cohort of limited cutaneous SSc patients without extensive ILD, we observed lower serum IgG levels, and higher β2-microglobulin, sBCMA, sCD23 and sCD27 levels in patients with pulmonary arterial hypertension (PAH). BAFF levels strongly correlated with Nt-pro-BNP levels, FVC/DLCO ratio and peak TRV in SSc-PAH patients. Cultured SSc B cells showed increased production of various angiogenic factors (angiogenin, angiopoietin-1, VEGFR-1, PDGF-AA, MMP-8, TIMP-1, L-selectin) and decreased production of angiopoietin-2 compared to HC.

**Conclusion:**

Soluble markers of B cell activation could be relevant tools to assess organ involvements, activity and severity in SSc. Their associations with PAH could plead for a role of B cell activation in the pathogenesis of pulmonary microangiopathy. B cells may contribute to SSc vasculopathy through production of angiogenic mediators.

## Introduction

Systemic sclerosis (SSc) is one of the most severe systemic autoimmune diseases (1). It is characterized by a clinical triad that combines immunological anomalies (autoantibodies, hyper-gammaglobulinemia, elevated acute phase reactants), fibrosing manifestations (in the skin, lungs and digestive tract) and vascular complications (such as pulmonary hypertension (PH), Raynaud phenomenon and digital ulcers) (2). SSc is a heterogeneous condition, whose burden on patient quality of life is variable and can range from mild symptoms to life-threatening situations (3–5). As such, there is an unmet need to develop tools that can better predict and diagnose severe organ involvements, as well as accurately assess disease activity and severity.

The pathophysiology of SSc reflects this clinical triad since it is currently seen as the result of the interactions between 3 major players: the immune system (aberrant activation of innate and adaptive immunity), fibroblasts (activation and acquisition of a myofibroblast phenotype) and the vasculature (obliterative microangiopathy with endothelial dysfunction) (6). Among the different immunity actors involved in SSc, the almost-constant presence of autoantibodies has long suggested a potential implication of B cells in the pathogenesis of the disease (7). Recent works have confirmed that B cells are activated in SSc, especially in early active forms of the disease, and contribute to fibrosis and vascular damage through production of pathogenic autoantibodies directed against endothelial cells and fibroblasts, pro-inflammatory and pro-fibrotic cytokines (8–10).

Interestingly, in other conditions involving B cells, several circulating proteins reflecting B cell activation have proved to be valuable diagnostic and prognostic markers (11). In patients with systemic *lupus erythematosus* (SLE), serum levels of BAFF (B-cell-activating factor) and APRIL (a proliferation-inducing ligand), 2 cytokines involved in the maturation and survival of B cells, as well as the soluble fraction of their receptors TACI (transmembrane activator and CAML interactor) and BCMA (B-cell maturation antigen), are elevated and correlate with disease activity (12–17). APRIL levels are also associated with the occurrence of lupus nephritis and may predict response to immunosuppressants (18). In patients with various types of B-cell lymphomas, serum concentrations of soluble CD23 (sCD23), sCD27 and sCD30 are elevated several years before diagnosis (19, 20). Circulating levels of CXCL13 (C-X-C motif chemokine 13), a chemokine known to attract B cells, are elevated, correlate with disease activity, severity and treatment response in patients with SLE, Sjögren’s syndrome, rheumatoid arthritis and ANCA-associated vasculitides (21–26). However, in the field of SSc, the relevance of such markers has not been extensively studied; and this was mostly done in ancient studies with small sample sizes (10, 11, 27–56).

To address this issue, we assessed the serum levels of several markers of B cell activation in a large and well-phenotyped SSc population, and tested their correlations with various disease characteristics including organs involvement, activity and severity. We discovered an association between B cell biomarkers and pulmonary arterial hypertension (PAH); and demonstrated that B cells can produce angiogenic mediators in SSc patients.

## Patients and methods

### Study population

All patients included were followed in the Lille National Referral Center for SSc and met the 2013 ACR/EULAR classification criteria for SSc (57). The cutaneous subset of SSc was defined as limited (lc) or diffuse (dc) according to LeRoy’s criteria (58). Early dcSSc was defined as dcSSc with disease duration < 3 years at the time of inclusion. Interstitial lung disease (ILD) was diagnosed on high-resolution CT-scan (HRCT); and staged as limited (if HRCT extent < 20% and forced vital capacity (FVC) ≥ 70%) or extensive (if HRCT extent > 20% or FVC < 70%) according to Goh’s system (59). PH was diagnosed by right-heart catheterization (RHC) using the 6^th^ World Symposium hemodynamic definitions and classified into causative groups according to the updated clinical classification of PH (60). Specifically, patients with PAH (= group 1 PH) fulfilled the hemodynamic definition for pre-capillary PH: mean pulmonary arterial pressure (mPAP) > 20 mmHg with pulmonary arterial wedge pressure (PAWP) ≤ 15 mmHg and pulmonary vascular resistance (PVR) ≥ 3 Wood units (WU).

In our first exploratory step, we designed our discovery cohort to be enriched in the most severe SSc complications, in order to unmask associations with biomarkers that could have been missed otherwise. To do so, we selected a panel of 80 patients from our clinical database, aiming at including approximately an equal proportion of patients with limited and diffuse cutaneous subsets, an equal proportion of patients with no/limited/extensive ILD, and about a quarter of patients with PH. For this population, there were no exclusion criteria.

In our second validation step focusing on PAH, we constituted an independent validation cohort, consisting of SSc patients with limited cutaneous subset, no extensive ILD, no treatment by immunosuppressants or corticosteroids; and either a diagnosis of group 1 PH (PAH) or no evidence of PH on screening exams. These criteria were chosen so that our biomarker assessment would not be biased by any active skin or lung involvement, nor by the effect of immunosuppressive drugs. For this population, patients with dcSSc, extensive ILD and/or treatment by immunosuppressants or corticosteroids were excluded.

### Data and samples collection

Data were collected systematically by a physician in a standardized case-report form for all patients referred to our day-patient clinic. All of them underwent a comprehensive evaluation performed within the same day. Data collected included patient and SSc characteristics, clinical parameters (including modified Rodnan skin score (mRSS) and New York Heart Association (NYHA) functional class), biological results (including C-reactive protein (CRP) and N-terminal prohormone of brain natriuretic peptide (Nt-pro-BNP) levels), transthoracic echocardiography (TTE) (including peak tricuspid regurgitation velocity (TRV) and left ventricle ejection fraction (LVEF)), pulmonary function tests (PFT) (including forced vital capacity (FVC), total lung capacity (TLC) and diffusing capacity of the lung for carbon monoxide (DLCO)), composite scores (European Scleroderma Study Group Activity Index (EScSG-AI) (61), Medsger severity score (62), scleroderma Health Assessment Questionnaire (sHAQ) (63)) and treatments. For patients with PAH, hemodynamic data at PAH diagnosis as well as from the last available RHC evaluation were also collected.

Whole blood samples were collected during routine venipuncture on the same day as clinical evaluation and were processed immediately after collection. Serum samples were obtained after clotting and centrifugation and were immediately stored at -80°C. Peripheral blood mononuclear cells (PBMCs) were isolated with density gradient centrifugation using Ficoll medium (Eurobio^©^), freezed in dimethyl sulfoxide-containing cryopreservative medium and stored in liquid nitrogen.

### Serum levels of soluble markers of B cell activation

A panel of circulating proteins reflecting immune activation, especially associated with B cells, was selected based on data from the literature. Serum concentrations of β2-microglobulin were measured using commercially available assays on the fully automated *Optilite^®^* turbidimetric analyzer (The Binding Site Group Ltd). Serum concentrations of rheumatoid factor (RF) were measured using commercially available fluorimetric assays (*Phadia 250^®^*, Thermo Fischer Scientific). Serum concentrations of IgA, IgG and IgM were measured using commercially available nephelemetric assays (Siemens). Normal range values were defined according to the manufacturer’s specifications. Serum concentrations of sBCMA and sCD21 were measured in duplicate using commercial ELISA assays (*Human BCMA/TNFRSF17 DuoSet ELISA*, cat. #DY193; *Human CD21 DuoSet ELISA*, cat. #DY4909-05). Serum concentrations of APRIL, BAFF, sTACI, CXCL13, sCD23, sCD25 and sCD27 were measured in duplicate using commercial customized pre-mixed multiplex assays (*Luminex Assay*, R&D Systems). All experiments were conducted according to the manufacturer’s protocol.

### B cell isolation and culture

PBMCs were thawed and washed in complete medium (Roswell Park Memorial Institute (RPMI) 1640 medium containing 10% heat-inactivated fetal calf serum (FCS), 100 UI/mL penicillin, 100 µg/mL streptomycin, 2 mM Glutamax^®^, and 1 mM pyruvate). B cells were isolated from PBMCs using a negative magnetic bead-assisted sorting assay (*EasySep Human Pan-B Cell Enrichment Kit*, cat. #19554, StemCell Technologies) according to the manufacturer’s protocol. Expression of a B cell marker (CD19) was analyzed by flow cytometry to assess the purity of sorted B cells. Purity (*i.e* proportion of CD19+ cells) was higher than 93% in all samples (**Supplemental Figure 1**).

Immediately after sorting, purified B cells were seeded on a 96-well U-bottom plate (200 000 B cells/mL, i.e. 40 000/well) within complete medium. They were cultured during 48 hours at 37°C in humidified atmosphere with 5% CO2; and either stimulated with anti-B cell receptor (BCR) 10 µg/mL (cat. #109-006-064, Jackson Immuno Research), CpG 10μg/mL (cat. #tlrl-2006-1, *In vivo*gen), CD40L-his 50ng/mL (cat. #2706-CL, R&D Systems) with anti-his crosslinking antibody 5µg/mL (cat. #MAB050, R&D Systems), and BAFF 100 ng/mL (cat. #7537-BF, R&D Systems); or left without stimulation. After culture, supernatants were collected and immediately stored at -80°C.

### B cell production of angiogenic mediators

A panel of proteins associated with angiogenesis was selected based on data from the literature, and included angiogenin, angiopoietin 1, angiopoietin 2, angiopoietin-like protein 6 (ANGPTL-6), Tie-2, endostatin, endothelin-1, vascular endothelial growth factor (VEGF), VEGF receptor (VEGFR)-1, VEGFR-2, VEGFR-3, platelet derived growth factor (PDGF)-AA, PDGF-AB, PDGF-BB, L-selectin, matrix metallopeptidase (MMP)-3, MMP-8, MMP-9, tissue inhibitor of metalloproteinases (TIMP)-1, bone morphogenetic protein (BMP)-9, thrombospondin 2, uPAR, and neuropilin 1. B cell production of these angiogenic mediators was assessed in culture supernatants in duplicate using commercial customized pre-mixed multiplex assays (*Luminex Assay*, R&D Systems), according to the manufacturer’s protocol.

### Statistical analyses

#### Description of the populations

Quantitative variables were expressed as means (± standard deviation, SD) in the case of normal distribution or medians (first quartile, Q1; third quartile, Q3) otherwise. Normality of distributions was assessed using histograms and the Shapiro-Wilk test. Categorical variables were expressed as numbers (percentage).

#### B cell biomarkers associations in the discovery population

In a first discovery step, associations between preselected quantitative B cell biomarkers and the presence of SSc were tested using analysis of covariance with an adjustment for age and gender (after log-transformation for normalization of distribution). The Cohen *d* (standardized adjusted difference between the 2 groups “SSc patients” and “healthy controls”) was computed as effect size with 95% confidence interval (CI). For this analysis, RF was considered as binary biomarker (positive/negative); and we used a multivariable logistic regression with RF biomarker as dependent variable and the status (“SSc patient”/”healthy control (HC)”), age and gender as independent variables. The effect size was assessed by the odds ratio (OR) with 95% CI for the risk of positive RF (with the status “HC” as reference value). All *p*-values were corrected for multiplicity using the Benjamini Hochberg procedure (False Discovery Rate) with a cut-off of 5%.

Exploratory analyses were performed among the “SSc patients” group to investigate the potential associations between B cell biomarkers and 13 prespecified disease characteristics. All analyses were adjusted for age, gender and immunosuppressive treatments. Given the nature exploratory of these analyses, no correction for multiplicity were performed. For the quantitative biomarkers, we used non-parametric correlation analysis or analysis of covariance, depending on the type of disease characteristics (quantitative or categorical). Effect sizes were assessed by the partial Spearman correlation coefficient ρ for quantitative characteristics, the adjusted Cohen *d* for binary characteristics and partial η^2^ statistics for categorical characteristics (64). The partial η^2^ is the proportion of variation accounted for by the characteristic being tested, after adjustment for all others. For the binary biomarker (RF), we used multivariable logistic regressions; and OR with 95% CI were computed as effect sizes.

Effect sizes were interpreted as follows. For Cohen *d*, values between 0.20-0.49 represent a small change, 0.50-0.79 a medium change, and ≥ 0.80 a large change. For the partial correlation coefficient ρ, values between 0.00-0.19 are very weak, 0.20-0.39 weak, 0.40-0.59 moderate, 0.60-0.79 strong, and ≥ 0.80 very strong. For partial η^2^, values of 0.01 are small, 0.06 medium, 0.14 large.

#### B cell biomarkers associations in the validation population

In a second validation step, we compared serum levels of B cell biomarkers between SSc patients with (n=18) and without (n=18) PAH using Mann-Whitney tests (given the small sample sizes) and computed the Cohen *d* effect sizes with 95% CI. Associations between B cell biomarkers and 3 prespecified disease characteristics (Nt-pro-BNP levels, peak TRV and FVC/DLCO ratio) were investigated among SSc-PAH patients using non-parametric correlation analysis with adjustment for age. Effect sizes were assessed by partial Spearman correlation coefficients ρ with 95% CI. For these analyses, RF was considered a quantitative variable. Given the nature exploratory of these analyses, no correction for multiplicity were performed.

#### B cell production of angiogenic mediators

In a third mechanistic step, we compared levels of several angiogenic mediators in B cell culture supernatants from HC (n=9) and SSc patients (n=18) using Mann-Whitney tests (given the small sample sizes) and computed the Cohen *d* effect sizes with 95% CI. Angiogenic mediators that were significantly different at the *p*=0.05 level between HC and SSc patients were compared between SSc patients with (n=9) and without (n=9) PAH. Given the nature exploratory of these analyses, no correction for multiplicity were performed.

#### Softwares

All statistical analyses were performed with the SAS software v9.4 (SAS Institute Inc.). Figures were created using GraphPad Prism v9.3.1 (GraphPad Software).

## Results

### Soluble markers of B cell activation are differentially expressed in SSc patients and correlate with several disease characteristics, including pulmonary hypertension

In a first exploratory step, we have determined the clinical and pathophysiological relevance of 14 soluble markers of B cell activation (RF, β2-microglobulin, IgG, IgA, IgM, BAFF, APRIL, sBCMA, sTACI, sCD21, sCD23, sCD25, sCD27, CXCL13) in SSc. We have selected a discovery panel of 80 SSc patients encompassing the whole spectrum of SSc clinical manifestations, measured the serum concentrations of these 14 biomarkers in this population, and compared them to those of 80 healthy blood donors (female 70%, mean age 43 ± 3 years).

The main characteristics of our discovery population are displayed in **Table 1** and can be summarized as follows: female 81%, mean age 56 ± 13 years; 41 lcSSc and 15 early dcSSc; 29 extensive and 25 limited ILD; and 19 PH among which 13 were group 1 PH (PAH). Conditions associated with RF positivity in the 44 RF-positive SSc patients were detailed in **Supplemental Table 1**. Overlap syndrome with another connective tissue disease was noted in 32% of cases, which seemed higher than previously reported (65–68): this could be explained by the enriched proportion of SSc-ILD patients in our population (67), a complication associated with overlap syndrome, and by systematic CTD screenings routinely performed during patient follow-up in our center. Among the 80 SSc patients, 7 had been treated by rituximab (RTX); and 3 of them received their last infusion less than 12 months before inclusion (in others, treatment was stopped several years prior). Detailed information regarding these 7 patients and their RTX regimens are provided in **Supplemental Table 2**.

**Table 1:** Characteristics of SSc patients from the discovery population.

	N	Value
**Demographic data**		
Females, n (%)	80	65 (81)
Age at inclusion (years), mean ± SD	80	56 ± 13
BMI (kg/m^2^), mean ± SD	80	24 ± 5
**Diagnosis of SSc**		
SSc subtype	80	
Early dcSSc, n (%)		15 (19)
Late dcSSc, n (%)		24 (30)
lcSSc, n (%)		41 (51)
Immunological profile	80	
Anti-centromere antibodies, n (%)		20 (25)
Anti-topoisomerase I antibodies, n (%)		21 (26)
Anti-RNA polymerase III antibodies, n (%)		7 (9)
Other antibody specificities, n (%)		17 (21)
Positive ANA without antibody specificity, n (%)		6 (8)
Negative ANA		9 (11)
Overlap with another connective tissue disease	79	25 (32)
Systemic *lupus erythematosus*, n (%)		4 (5)
Sjogren’s syndrome, n (%)		12 (15)
Inflammatory myopathy, n (%)		4 (5)
Rheumatoid arthritis, n (%)		2 (3)
Lymphoma (current or previous history), n (%)	80	0 (0)
Disease duration at inclusion		
Since diagnosis (years), mean ± SD	80	10 ± 10
Since first non-Raynaud symptom (years), mean ± SD	78	11 ± 10
Since Raynaud phenomenon onset (years), mean ± SD	76	14 ± 13
**History of organ involvements**		
Interstitial lung disease	80	
No ILD, n (%)		26 (33)
Limited ILD, n (%)		25 (31)
Extensive ILD, n (%)		29 (36)
ILD duration at inclusion (years), mean ± SD	54	7 ± 7
Pulmonary hypertension, n (%)	80	19 (24)
Group 1, n (%)		13 (17)
Group 1’, n (%)		1 (1)
Group 2, n (%)		6 (8)
Group 3, n (%)		3 (4)
PH duration at inclusion (years), mean ± SD	19	4 ± 4
Scleroderma renal crisis, n (%)	80	0 (0)
History of digital ulcers, n (%)	80	49 (61)
**Clinical evaluation at inclusion**		
Modified Rodnan skin score, mean ± SD	79	7.6 ± 6.5
in dcSSc patients, mean ± SD	41	10.8 ± 6.8
NYHA functional class	80	
Class I, n (%)		29 (36)
Class II, n (%)		21 (26)
Class III, n (%)		23 (29)
Class IV, n (%)		7 (9)
6-minute walk distance (m), mean ± SD	74	401 ± 117
6-minute walk distance (% predicted), mean ± SD	74	72 ± 18
Active Raynaud attacks at inclusion, n (%)	76	35 (46)
Active digital ulcers at inclusion, n (%)	80	16 (20)
Telangiectasias, n (%)	79	59 (75)
Calcinosis cutis, n (%)	74	12 (16)
**Biological data**		
ESR (mm/h), mean ± SD	74	17 ± 19
CRP (mg/L), mean ± SD	80	6.9 ± 8.9
Creatinin (mg/L), mean ± SD	80	7.9 ± 2.4
Estimated GFR (mL/min/1,73m^2^), mean ± SD	80	94 ± 27
Nt-pro-BNP (pg/mL), mean ± SD	80	578 ± 1452
Ferritin (ng/mL), mean ± SD	79	112 ± 121
Uric acid (mg/L), mean ± SD	80	47 ± 15
Complement activation, n (%)	80	1 (1)
**Transthoracic echocardiography**		
Left ventricular ejection fraction (%), mean ± SD	79	64 ± 7
Left ventricular diastolic dysfunction, n (%)	78	62 (80)
Peak TRV (m/s), mean ± SD	69	2.78 ± 0.68
RA area (cm^2^), mean ± SD	67	15 ± 5
**Pulmonary function tests**		
TLC (% predicted), mean ± SD	78	83 ± 19
FVC (% predicted), mean ± SD	80	88 ± 23
DLCO (% predicted), mean ± SD	77	57 ± 20
KCO (% predicted), mean ± SD	78	73 ± 21
**Composite scores**		
EScSG-AI score, mean ± SD	78	1.58 ± 1.31
in lcSSc patients, mean ± SD	41	1.46 ± 1.22
in dcSSc patients, mean ± SD	37	1.72 ± 1.40
Medsger severity score, mean ± SD	77	5.90 ± 2.86
sHAQ score, mean ± SD	72	0.90 ± 0.63
**Treatments at inclusion**		
Corticosteroids, n (%)	80	47 (59)
Corticosteroid dosage (mg prednisone equivalent/day), mean ± SD	47	8 ± 3
Immunosuppressants, n (%)	80	43 (54)
Cyclophosphamide, n (%)		0 (0)
Mycophenolate mofetil, n (%)		31 (39)
Methotrexate, n (%)		3 (4)
Azathioprine, n (%)		2 (3)
Rituximab (current or previous), n (%)		7 (9)
Current treatment by rituximab (last infusion < 12 months)		3 (4)
Hydroxychloroquine, n (%)	80	7 (8.8)
PAH specific drugs*, n (%)	80	21 (26)

ANA, antinuclear antibodies; BMI, body mass index; CRP, C-reactive protein; dc, diffuse cutaneous; DLCO, diffusing capacity of the lung for carbon monoxide; EScSG-AI, European Scleroderma Study Group Activity Index; ESR, erythrocyte sedimentation rate; FVC, forced vital capacity; GFR, glomerular filtration rate; sHAQ, scleroderma Health Assessment Questionnaire; ILD, interstitial lung disease; KCO, diffusing coefficient for carbon monoxide; lc, limited cutaneous; Nt-pro-BNP, N-terminal prohormone of brain natriuretic peptide; NYHA, New York Heart Association; PAH, pulmonary arterial hypertension; RA, right atrium; SSc, systemic sclerosis; TLC, total lung capacity; TRV, tricuspid regurgitation velocity.

*PAH specific drugs included phosphodiesterase 5 inhibitors, endothelin receptor antagonists and prostacyclin analogues. These could have been prescribed for PAH, digital ulcers and/or refractory Raynaud phenomenon.

**Figure 1:**
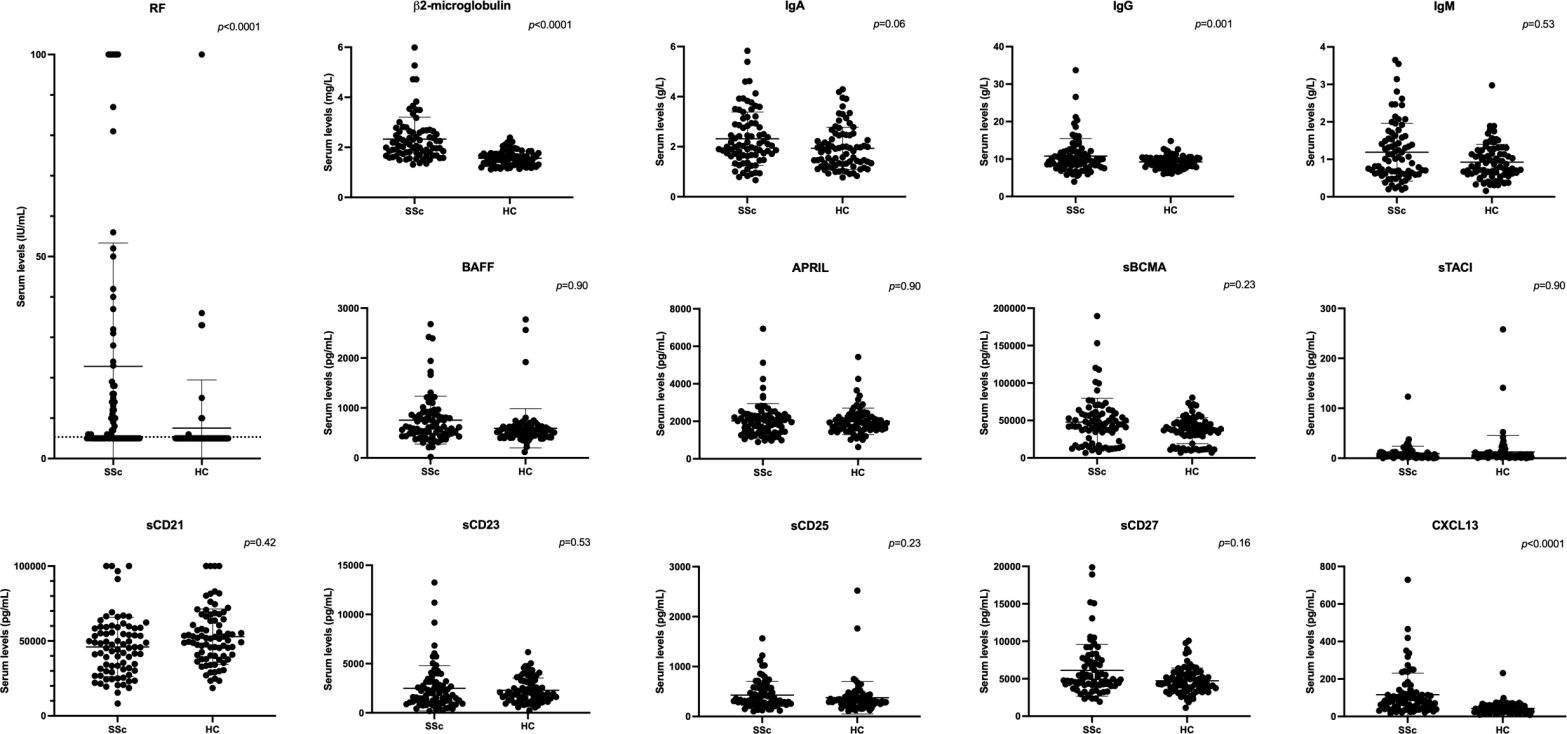
Serum levels of soluble markers of B cell activation in SSc patients and healthy controls (discovery cohort). APRIL, a proliferation-inducing ligand; BAFF, B-cell-activating factor; BCMA, B-cell maturation antigen; CD, cluster of differentiation; CXCL13, C-X-C motif chemokine 13; HC, healthy controls; Ig, immunoglobulin; RF, rheumatoid factor; s, soluble; SSc, systemic sclerosis; TACI, transmembrane activator and CAML interactor. The dotted line on the RF panel represents the threshold for RF positivity. Error bars display means and standard deviations. *p*-values are adjusted for age, gender and multiplicity. The *p*-value displayed on the RF panel refers to the analysis using RF as a binary variable.

After adjustment on age, gender and multiplicity, we observed a significantly increased proportion of RF positivity (55% *vs.* 10%, *p*<0.0001) and higher median levels of β2-microglobulin (2.09 [1.76;2.64] *vs.* 1.55 [1.34;1.74] mg/L, *p*<0.0001), IgG (9.66 [8.30;12.04] *vs.* 9.30 [7.96;10.31] g/L, *p*=0.001) and CXCL13 (81.73 [46.58;120.5] *vs.* 36.95 [24.46;55.77] pg/mL, *p*<0.0001) in SSc patients compared with HC (**Table 2**; **Figure 1**). There was also a trend for an increase in IgA concentrations (2.03 [1.64;2.91] *vs.* 1.82 [1.31;2.45] g/L, *p*=0.06) in SSc patients. There was no difference in serum levels of IgM, BAFF, APRIL, sBCMA, sTACI, sCD21, sCD23, sCD25 and sCD27 between the 2 groups. A sensitivity analysis excluding the 7 patients that received RTX yielded similar results, except that the increase in serum IgA levels in the SSc patient group reached statistical significance (*p*=0.04) (**Supplemental Table 3**).

**Table 2:** Serum levels of soluble markers of B cell activation in the SSc patients and healthy controls (discovery cohort).

Biomarkers	Healthy controls (N=80)	SSc patients (N=80)	Effect size^1^	*p*-values^2^	*p*-values adjusted for FDR^3^
**Positive RF**, n (%)	8 (10%)	44 (55%)	12.68 (4.78; 33.61)	<0.0001	**<0.0001**
**β2-microglobulin** (mg/L), median (Q1;Q3)	1.55 (1.34; 1.74)	2.09 (1.76; 2.64)	0.61 (0.35; 0.87)	<0.0001	**<0.0001**
**IgA** (g/L), median (Q1;Q3)	1.82 (1.31; 2.45)	2.03 (1.64; 2.91)	0.36 (0.05; 0.68)	0.02	0.06
**IgG** (g/L), median (Q1;Q3)	9.30 (7.96; 10.31)	9.66 (8.30; 12.04)	0.55 (0.25; 0.86)	0.0004	**0.001**
**IgM** (g/L), median (Q1;Q3)	0.81 (0.61; 1.20)	1.01 (0.62; 1.57)	0.12 (-0.17; 0.41)	0.42	0.53
**BAFF** (pg/ml), median (Q1;Q3)	534 (446; 624)	620 (478; 864)	0.02 (-0.28; 0.32)	0.90	0.90
**APRIL** (pg/ml), median (Q1;Q3)	1911 (1619; 2236)	1958 (1425; 2313)	0.03 (-0.28; 0.34)	0.83	0.90
**sBCMA** (pg/ml), median (Q1;Q3)	37344 (29070; 47014)	44019 (24470; 59028)	0.24 (-0.07; 0.55)	0.13	0.23
**sTACI** (pg/ml), median (Q1;Q3)	3.79 (1.64; 7.18)	5.05 (1.79; 10.62)	0.24 (-0.07; 0.55)	0.14	0.90
**sCD21** (pg/ml), median (Q1;Q3)	51516 (39990; 64006)	45520 (30635; 58414)	-0.17 (-0.47; 0.13)	0.27	0.42
**sCD23** (pg/ml), median (Q1;Q3)	1952 (1405; 3168)	1803 (984.8; 3144)	-0.13 (-0.44; 0.18)	0.40	0.53
**sCD25** (pg/ml), median (Q1;Q3)	305 (246; 391)	327.7 (248; 569)	0.24 (-0.07; 0.55)	0.13	0.23
**sCD27** (pg/ml), median (Q1;Q3)	4440 (3615; 5658)	4906 (4191; 7395)	0.28 (-0.02; 0.59)	0.07	0.16
**CXCL13** (pg/ml), median (Q1;Q3)	36.95 (24.46; 55.77)	81.73 (46.58; 120.5)	1.01 (0.69; 1.33)	<0.0001	**<0.0001**

APRIL, a proliferation-inducing ligand; BAFF, B-cell-activating factor; BCMA, B-cell maturation antigen; CD, cluster of differentiation; CXCL13, C-X-C motif chemokine 13; FDR, false discovery rate; Ig, immunoglobulin; Q, quartile; RF, rheumatoid factor; s, soluble; SSc, systemic sclerosis; TACI, transmembrane activator and CAML interactor.

Results are expressed as median (first quartile; third quartile) for quantitative biomarkers and as frequency (percentage) otherwise.

All analyses were adjusted for age and gender.

^1^ For quantitative biomarkers, effect sizes were calculated on log transformed variables using the Cohen d. Absolute values of 0.20–0.49 represent a small change; values of 0.50–0.79 a medium change; and values of ≥ 0.80 a large change. For the binary biomarker, effect size is the odds ratio of the status for the risk of positive RF with the status control as reference value.

^2^ p-values calculated on log-transformed variables for quantitative biomarkers.

^3^ p-values corrected for multiplicity using the False Discovery Rate (FDR) method (Benjamini Hochberg procedure).

As previous studies reported elevated serum BAFF levels in SSc patients (10, 29, 31, 33–36), we tried to further investigate the association between BAFF concentrations and disease status. In univariate analysis, the difference in circulating BAFF levels between the 2 groups reached statistical significance (620 [478;864] *vs.* 534 [446;624] pg/mL in SSc patients and HC respectively, *p*=0.04). However, this was no longer the case after adjustment on age and gender (*p*=0.90): indeed, there was a positive association between age and BAFF concentrations in our population (*r*=0.17, *p*=0.03). As none of the HC were under immunosuppressants, we also wondered immunosuppressive therapy in SSc patients could contribute explain our result; however we did not observe any difference in the serum BAFF levels of HC when compared to SSc patients with (534 [446;624] *vs.* 630 [434;887] pg/mL respectively, *p=*0.80) or without (534 [446;624] *vs.* 586 [487;859] pg/mL respectively, *p=*0.53) immunosuppressants.

**Figure 2:**
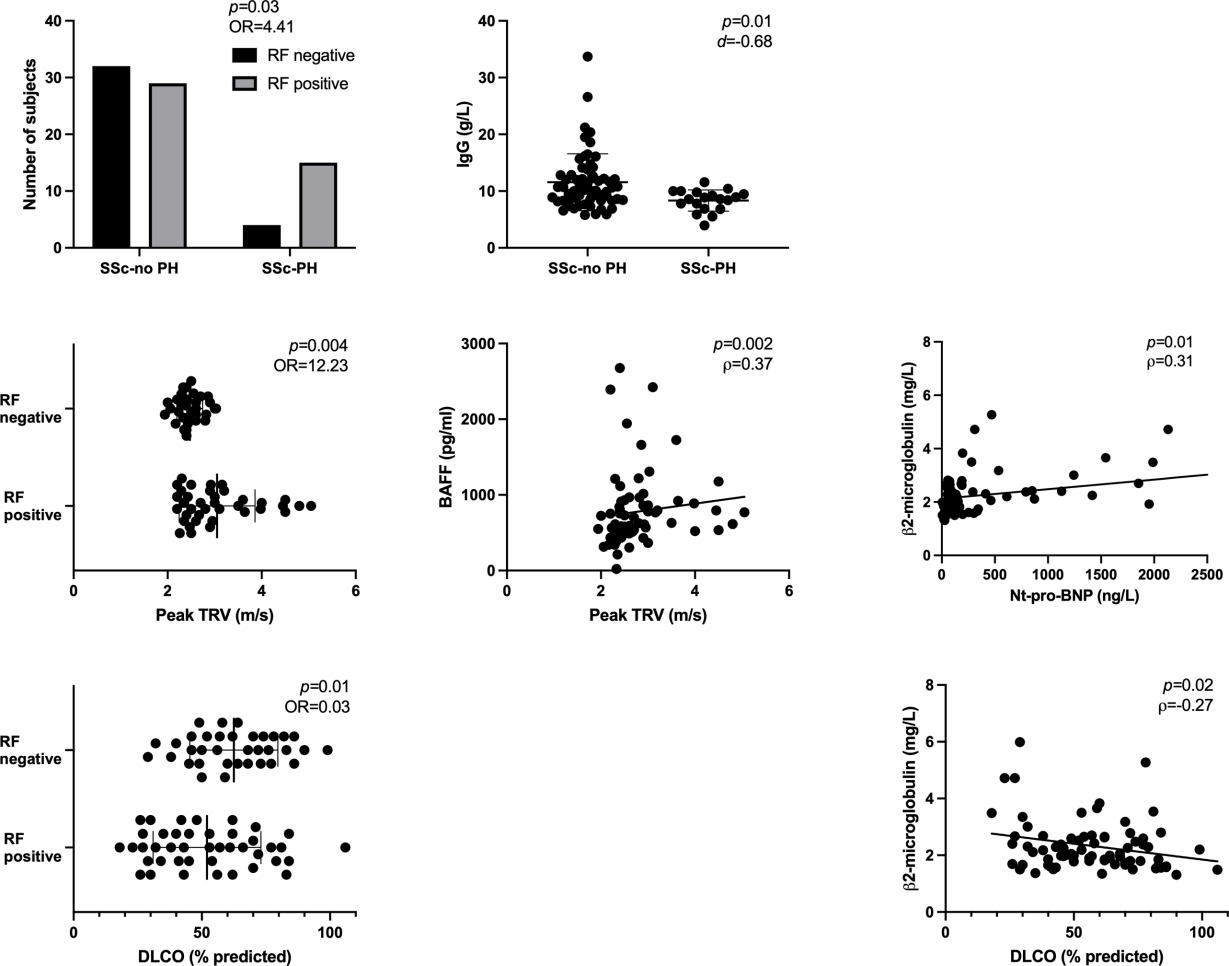
Significant associations between serum levels of soluble markers of B cell activation and SSc characteristics related to PH, assessed among the 80 SSc patients from the discovery cohort. BAFF, B-cell-activating factor; DLCO, diffusing capacity of the lung for carbon monoxide; Ig, immunoglobulin; Nt-pro-BNP, N-terminal prohormone of brain natriuretic peptide; OR, odds ratio; PH, pulmonary hypertension; RF, rheumatoid factor; SSc, systemic sclerosis; TRV, tricuspid regurgitation velocity. Error bars display means and standard deviations. *p*-values are adjusted for age, gender and immunosuppressive drugs.

Next, we assessed the associations between serum levels of soluble markers of B cell activation and various disease characteristics in the SSc patient group (**Table 3**). After adjustment on age, gender and immunosuppressive drugs, significant associations were identified between B cell biomarkers and SSc parameters related to:

- disease phenotype: SSc subtype with RF positivity (*p*=0.004) and sCD23 (*p*=0.03); autoantibody profile with IgG (*p*<0.001) and APRIL (*p*=0.02);
- global disease activity and severity: CRP with β2-microglobulin (*p*=0.03), BAFF (*p*=0.001), APRIL (*p*=0.002), sCD21 (*p*=0.02) and sCD25 (*p*=0.03); EScSG-AI score with sCD21 (*p*=0.02); total Medsger score with β2-microglobulin (*p*=0.01);
- ILD: history of ILD with APRIL (*p*=0.02) and sBCMA (*p*=0.02); FVC with IgA (*p*=0.002) and with IgG (*p*=0.03);
- PH (**Figure 2**): history of PH with RF positivity (*p*=0.03) and IgG (*p*=0.01); Nt-pro-BNP with β2-microglobulin (*p*=0.01); peak TRV with RF positivity (*p*=0.004) and BAFF (*p*=0.002); DLCO with RF positivity (*p*=0.01) and β2-microglobulin (*p*=0.02). There was also a trend for associations between Nt-pro-BNP and sBCMA (*p*=0.07), DLCO and BAFF (*p*=0.09) and DLCO and sCD23 levels (*p*=0.07).

**Table 3:** Associations between serum levels of soluble markers of B cell activation and disease characteristics assessed among the 80 SSc patients from the discovery cohort.

	Positive RF^1^	β2m^5^(mg/L)	IgA^5^(g/L)	IgG^5^(g/L)	IgM^5^(g/L)	BAFF^5^(pg/mL)	APRIL^5^(pg/mL)	sBCMA^5^(pg/mL)	sTACI^5^(pg/mL)	sCD21^5^(pg/mL)	sCD23^5^(pg/mL)	sCD25^5^(pg/mL)	sCD27^5^(pg/mL)	CXCL13^5^(pg/mL)
**SSc subtype** lc *vs* dc	**p=0.004** OR=5.18 (1.72;15.63)	p=0.72 d=0.07 (-0.31;0.44)	p=0.53 d=0.14 (-0.30;0.58)	p=0.98 d=0.01 (-0.40;0.42)	p=0.73 d=-0.07 (-0.45;0.31)	p=0.91 d=-0.02 (-0.45;0.40)	p=0.79 d=0.06 (-0.37;0.48)	p=0.84 d=-0.04 (-0.46;0.37)	p=0.17 d=0.30 (-0.13;0.74)	p=0.15 d=0.30 (-0.11;0.72)	**p=0.03** d=0.46 (0.04;0.89)	p=0.23 d=-0.27 (-0.71;0.17)	p=0.08 d=-0.39 (-0.83;0.04)	p=0.22 d=0.27 (-0.16;0.71)
**mRSS**^2^	p=0.29 OR=0.75 (0.44;1.27)	p=0.50 ρ=-0.08 (-0.30;0.15)	p=0.67 ρ=-0.05 (-0.27;0.18)	p=0.21 ρ=-0.14 (-0.36;0.09)	p=0.51 ρ=0.08 (-0.15;0.30)	p=0.12 ρ=0.18 (-0.05;0.39)	p=0.16 ρ=0.16 (-0.06;0.37)	p=0.56 ρ=-0.07 (-0.29;0.16)	p=0.79 ρ=-0.03 (-0.25;0.20)	p=0.66 ρ=-0.05 (-0.27;0.18)	p=0.36 ρ=-0.11 (-0.32;0.12)	p=0.65 ρ=0.05 (-0.18;0.27)	p=0.98 ρ=-0.003 (-0.23;0.22)	p=0.87 ρ=-0.02 (-0.24;0.21)
**Disease duration**^2^ (years)	p=0.90 OR=1.03 (0.65;1.64)	p=0.62 ρ=-0.06 (-0.28;0.17)	p=0.70 ρ=-0.04 (-0.27;0.18)	p=0.57 ρ=-0.07 (-0.28;0.16)	p=0.85 ρ=0.02 (-0.20;0.24)	p=0.42 ρ=0.09 (-0.14;0.31)	p=0.33 ρ=-0.11 (-0.33;0.11)	p=0.78 ρ=-0.03 (-0.25;0.19)	p=0.44 ρ=-0.09 (-0.31;0.14)	p=0.78 ρ=-0.03 (-0.25;0.19)	p=0.71 ρ=0.04 (-0.18;0.26)	p=0.88 ρ=-0.02 (-0.24;0.21)	p=0.90 ρ=-0.01 (-0.24;0.21)	p=0.41 ρ=-0.10 (-0.31;0.13)
**Auto-Ab** ATA *vs* ACA ARA *vs* ACA other *vs* ACA	p=0.46 OR 1.90 (0.42;8.53) OR 5.12 (0.68;38.46) OR 2.11 (0.48;9.25)	p=0.63 η^2 =^ 0.03 (0.00;0.10)	p=0.23 η^2 =^ 0.06 (0.00;0.16)	**p<0.0001** η^2 =^ 0.29 (0.09;0.42)	p=0.82 η^2 =^ 0.01 (0.00;0.06)	p=0.64 η^2 =^ 0.03 (0.00;0.09)	**p=0.02** η^2 =^ 0.14 (0.00;0.26)	p=0.40 η^2 =^ 0.04 (0.00;0.13)	p=0.62 η^2 =^ 0.03 (0.00;0.10)	p=0.51 η^2 =^ 0.03 (0.00;0.11)	p=0.40 η^2 =^ 0.04 (0.00;0.13)	p=0.64 η^2 =^ 0.03 (0.00;0.09)	p=0.19 η^2 =^ 0.07 (0.00;0.17)	p=0.24 η^2 =^ 0.06 (0.00;0.16)
**ILD** lim *vs* none ext *vs* none	p=0.18 OR 0.94 (0.28;3.14) OR 0.34 (0.09;1.23)	p=0.70 η^2 =^ 0.01 (0.00;0.07)	p=0.10 η^2 =^ 0.06 (0.00;0.17)	p=0.36 η^2 =^ 0.03 (0.00;0.11)	p=0.96 η^2 =^ 0.001 (0.00;0.01)	p=0.16 η^2 =^ 0.05 (0.00;0.15)	**p=0.02** η^2 =^ 0.10 (0.00;0.22)	**p=0.02** η^2 =^ 0.10 (0.00;0.22)	p=0.76 η^2 =^ 0.01 (0.00;0.06)	p=0.17 η^2 =^ 0.05 (0.00;0.15)	p=0.89 η^2 =^ 0.003 (0.00;0.04)	p=0.20 η^2 =^ 0.04 (0.00;0.14)	p=0.11 η^2 =^ 0.06 (0.00;0.16)	p=0.36 η^2 =^ 0.03 (0.00;0.11)
**FVC**^4^ (%)	p=0.38 OR=0.39 (0.05;3.15)	p=0.05 ρ=-0.22 (-0.42;0.004)	**p=0.002** ρ=-0.35 (-0.53;-0.14)	**p=0.03** ρ=-0.25 (-0.45;-0.03)	p=0.59 ρ=-0.06 (-0.28;0.16)	p=0.99 ρ=-0.001 (-0.22;0.22)	p=0.94 ρ=-0.01 (-0.23;0.22)	p=0.56 ρ=-0.07 (-0.29;0.16)	p=0.16 ρ=-0.16 (-0.37;0.06)	p=0.67 ρ=0.05 (-0.18;0.27)	p=0.79 ρ=-0.03 (-0.25;0.20)	p=0.47 ρ=0.08 (-0.14;0.30)	p=0.66 ρ=0.05 (-0.18;0.27)	p=0.06 ρ=-0.22 (-0.42;0.01)
**PH** *vs* no PH	**p=0.03** OR=4.41 (1.15;16.92)	p=0.07 d=0.41 (-0.03;0.85)	p=0.97 d=-0.01 (-0.52;0.50)	**p=0.01** d=-0.68 (-1.17;-0.19)	p=0.87 d=-0.04 (-0.48;0.41)	p=0.82 d=0.06 (-0.44;0.56)	p=0.34 d=-0.24 (-0.74;0.25)	p=0.47 d=0.18 (-0.31;0.67)	p=0.90 d=-0.03 (-0.54;0.47)	p=0.07 d=0.46 (-0.03;0.94)	p=0.17 d=0.34 (-0.15;0.84)	p=0.36 d=-0.24 (-0.75;0.27)	p=0.14 d=-0.39 (-0.89;0.12)	p=0.65 d=0.12 (-0.39;0.63)
**Nt-pro-BNP**^3^ (pg/mL)	p=0.36 OR=1.20 (0.82;1.75)	**p=0.01** ρ=0.31 (0.09;0.50)	p=0.83 ρ=0.03 (-0.20;0.25)	p=0.96 ρ=0.01 (-0.22;0.23)	p=0.50 ρ=-0.08 (-0.30;0.15)	p=0.85 ρ=-0.02 (-0.24;0.20)	p=0.83 ρ=-0.02 (-0.25;0.20)	p=0.07 ρ=0.21 (-0.02;0.41)	p=0.46 ρ=0.09 (-0.14;0.30)	p=0.35 ρ=0.11 (-0.12;0.32)	p=0.28 ρ=0.13 (-0.10;0.34)	p=0.92 ρ=0.01 (-0.21;0.24)	p=0.85 ρ=-0.02 (-0.24;0.20)	p=0.29 ρ=0.12 (-0.11;0.34)
**Peak TRV**^3^ (m/s)	**p=0.004** OR=12.23 (2.23;66.90)	p=0.26 ρ=0.14 (-0.11;0.37)	p=0.48 ρ=-0.09 (-0.32;0.16)	p=0.27 ρ=-0.14 (-0.37;0.11)	p=0.22 ρ=0.15 (-0.09;0.38)	**p=0.002** ρ=0.37 (0.13;0.56)	p=0.40 ρ=0.10 (-0.14;0.34)	p=0.80 ρ=-0.03 (-0.27;0.21)	p=0.28 ρ=-0.14 (-0.36;0.11)	p=0.23 ρ=-0.15 (-0.38;0.10)	p=0.62 ρ=0.06 (-0.18;0.30)	p=0.98 ρ=-0.003 (-0.24;0.24)	p=0.31 ρ=-0.13 (-0.36;0.12)	p=0.95 ρ=-0.01 (-0.25;0.23)
**DLCO**^4^ (%)	**p=0.01** OR=0.03 (0.002;0.43)	**p=0.02** ρ=-0.27 (-0.47;-0.05)	p=0.21 ρ=-0.15 (-0.36;0.08)	p=0.74 ρ=0.04 (-0.19;0.26)	p=0.83 ρ=0.03 (-0.20;0.25)	p=0.09 ρ=-0.20 (-0.41;0.03)	p=0.90 ρ=-0.01 (-0.24;0.21)	p=0.96 ρ=-0.01 (-0.23;0.22)	p=0.87 ρ=0.02 (-0.21;0.25)	p=0.84 ρ=0.02 (-0.21;0.25)	p=0.07 ρ=-0.21 (-0.42;0.02)	p=0.44 ρ=0.09 (-0.14;0.31)	p=0.36 ρ=0.11 (-0.12;0.33)	p=0.15 ρ=-0.17 (-0.38;0.06)
**CRP**^2^ (mg/L)	p=0.68 OR=1.15 (0.59;2.28)	**p=0.03** ρ=0.25 (0.02;0.45)	p=0.51 ρ=-0.08 (-0.29;0.15)	p=0.76 ρ=0.03 (-0.19;0.26)	p=0.20 ρ=0.15 (-0.08;0.36)	**p=0.001** ρ=0.37 (0.15;0.54)	**p=0.002** ρ=0.35 (0.13;0.53)	p=0.67 ρ=-0.05 (-0.27;0.18)	p=0.81 ρ=0.03 (-0.20;0.25)	**p=0.02** ρ=-0.26 (-0.46;-0.04)	p=0.99 ρ=0.001 (-0.22;0.23)	**p=0.03** ρ=0.24 (0.02;0.44)	p=0.92 ρ=0.01 (-0.21;0.23)	p=0.85 ρ=0.02 (-0.20;0.25)
**EScSG-AI score**^2^	p=0.15 OR=2.12 (0.76;5.92)	p=0.12 ρ=0.18 (-0.05;0.39)	p=0.34 ρ=0.11 (-0.12;0.33)	p=0.85 ρ=-0.02 (-0.25;0.21)	p=0.30 ρ=0.12 (-0.11;0.34)	p=0.08 ρ=0.20 (-0.03;0.41)	p=0.38 ρ=0.10 (-0.13;0.32)	p=0.48 ρ=-0.08 (-0.30;0.15)	p=0.45 ρ=0.09 (-0.14;0.31)	**p=0.02** ρ=-0.28 (-0.47;-0.05)	p=0.84 ρ=-0.02 (-0.25;0.20)	p=0.17 ρ=0.16 (-0.07;0.37)	p=0.74 ρ=0.04 (-0.19;0.26)	p=0.07 ρ=0.21 (-0.02;0.42)
**Medsger score**^2^	p=0.97 OR=1.00 (0.85;1.18)	**p=0.01** ρ=0.29 (0.06;0.48)	p=0.57 ρ=0.07 (-0.16;0.29)	p=0.87 ρ=0.02 (-0.21;0.25)	p=0.74 ρ=0.04 (-0.19;0.26)	p=0.06 ρ=0.22 (-0.01;0.43)	p=0.40 ρ=0.10 (-0.13;0.32)	p=0.90 ρ=-0.01 (-0.24;0.21)	p=0.26 ρ=0.13 (-0.10;0.35)	p=0.64 ρ=0.06 (-0.18;0.28)	p=0.80 ρ=0.03 (-0.20;0.26)	p=0.91 ρ=0.01 (-0.22;0.24)	p=0.74 ρ=0.04 (-0.19;0.26)	p=0.20 ρ=0.15 (-0.08;0.37)

ACA, anti-centromere antibodies; ARA, anti-RNA polymerase III antibodies; ATA, anti-topoisomerase antibodies; APRIL, a proliferation-inducing ligand; BAFF, B-cell-activating factor; BCMA, B-cell maturation antigen; CD, cluster of differentiation; CRP, C-reactive protein; CXCL13, C-X-C motif chemokine 13; dc, diffuse cutaneous; DLCO, diffusing capacity of the lung for carbon monoxide; EScSG-AI, European Scleroderma Study Group Activity Index; ext, extensive; FVC, forced vital capacity; Ig, immunoglobulin; lc, limited cutaneous; lim, limited; mRSS, modified Rodnan skin score; Nt-pro-BNP, N-terminal prohormone of brain natriuretic peptide; PH, pulmonary hypertension; RF, rheumatoid factor; s, soluble; SD, standard deviation; SSc, systemic sclerosis; TACI, transmembrane activator and CAML interactor; TRV, tricuspid regurgitation velocity; β2m, β2-microglobulin.

All analyses are adjusted for age, gender and immunosuppressive treatments. Given the exploratory nature of this analysis, p-values were not corrected for multiplicity.

^1^ For associations between binary biomarkers (RF) and disease characteristics, effect sizes are expressed as odds ratio (OR) for the risk of positive RF (with 95% confidence interval).

^2^ OR per increasing of one unit in the log transformed variable.

^3^ OR per increasing of one unit in the original variable.

^4^ OR per increasing of 100 units in the original variables.

^5^ For associations between quantitative biomarkers and quantitative disease characteristics, effect sizes are expressed as the partial Spearman correlation coefficient ρ (with 95% confidence interval). The usual interpretation is that an absolute value of correlation between 0.00 to 0.19 is very weak; 0.20 to 0.39 weak; 0.40 to 0.59 moderate; 0.60 to 0.79 strong; and greater than 0.80 very strong.

For associations between quantitative biomarkers and binary disease characteristics, effect sizes are expressed as the Cohen d. Values of 0.20–0.49 represent a small change; values of 0.50–0.79 a medium change; and values of ≥ 0.80 a large change.

For associations between quantitative biomarkers and nominal disease characteristics, effect sizes are expressed by the partial η^2^ statistics proposed by Cohen. It is the partial proportion of variation accounted for by the effect being tested. Values of 0.01 are small; 0.06 medium; and 0.14 large.

Overall, these data suggest that soluble markers of B cell activation are differentially expressed in SSc patients and correlate with several disease characteristics, especially PH.

### Circulating levels of β2-microglobulin, IgG, sBCMA, sCD23 and sCD27 are differentially expressed in SSc patients with and without PAH; and serum BAFF levels correlate with clinical markers of PH in SSc-PAH patients

Since involvement of B cells in skin fibrosis, SSc-ILD and disease activity has already been reported (7), we chose to further focus on the association between B cell activation and SSc-PAH observed in our discovery population. We assessed the serum levels of the same B cell biomarkers in an independent validation cohort, consisting of SSc patients with limited cutaneous subset, no extensive ILD, no treatment by immunosuppressants or corticosteroids; and either a diagnosis of group 1 PH (PAH) confirmed by RHC (n=18) or no evidence of PH on screening exams (n=18) (**Table 4**). These inclusion criteria were chosen so that our biomarker assessment would not be biased by any active skin or lung involvement, nor by the effect of immunosuppressive drugs. Groups were matched on age (± 5 years) and gender.

**Table 4:** Characteristics of SSc-PAH and SSc-no PAH patients from the validation population.

	SSc-no PAH		SSc-PAH	
	N	Value	N	Value
**Demographic data**				
Females, n (%)	18	17 (94)	18	17 (94)
Age at inclusion (years), mean ± SD	18	70 ± 10	18	69 ± 9.3
BMI (kg/m^2^), mean ± SD	18	26 ± 5	18	25 ± 4.8
**Diagnosis of SSc**				
SSc subtype	18		18	
lcSSc, n (%)		18 (100)		18 (100)
dcSSc, n (%)		0 (0)		0 (0)
Immunological profile	18		18	
Anti-centromere antibodies, n (%)		18 (100)		17 (94)
Anti-topoisomerase I antibodies, n (%)		0 (0)		0 (0)
Anti-RNA polymerase III antibodies, n (%)		0 (0)		0 (0)
Anti-fibrillarin, n (%)		0 (0)		1 (6)
Overlap with another connective tissue disease	18	6 (33)	18	6 (33)
Systemic *lupus erythematosus*, n (%)		0 (0)		0 (0)
Sjogren’s syndrome, n (%)		6 (33)		6 (33)
Inflammatory myopathy, n (%)		0 (0)		0 (0)
Rheumatoid arthritis, n (%)		0 (0)		0 (0)
Lymphoma (current or previous history), n (%)	18	0 (0)	18	0 (0)
Disease duration at inclusion				
Since diagnosis (years), mean ± SD	18	14 ± 9	18	17 ± 9.8
Since first non-Raynaud symptom (years), mean ± SD	16	16 ± 7	17	18 ± 8.1
Since Raynaud phenomenon onset (years), mean ± SD	18	23 ± 11	18	29 ± 15
**History of organ involvements**				
Interstitial lung disease	18		18	
No ILD, n (%)		14 (73)		14 (73)
Limited ILD, n (%)		4 (22)		4 (22)
Extensive ILD, n (%)		0 (0)		0 (0)
ILD duration at inclusion (years), mean ± SD	4	7 ± 4	4	10 ± 11
Pulmonary hypertension, n (%)	18	0 (0)	18	18 (100)
Group 1, n (%)		0 (0)		18 (100)
Group 1’, n (%)		0 (0)		2 (15)
Group 2, n (%)		0 (0)		0 (0)
Group 3, n (%)		0 (0)		0 (0)
PH duration at inclusion (years), mean ± SD			18	3 ± 2.3
Scleroderma renal crisis, n (%)	18	0 (0)	18	0 (0)
History of digital ulcers, n (%)	18	11 (61)	18	12 (67)
**Clinical evaluation at inclusion**				
Modified Rodnan skin score, mean ± SD	18	4.1 ± 3.8	18	7.1 ± 6.1
NYHA functional class	18		18	
Class I, n (%)		8 (44)		4 (22)
Class II, n (%)		8 (44)		3 (17)
Class III, n (%)		1 (6)		7 (39)
Class IV, n (%)		1 (6)		4 (22)
6-minute walk distance (m), mean ± SD	14	383 ± 86	17	298 ± 125
6-minute walk distance (% predicted), mean ± SD	14	78 ± 20	17	59 ± 21
Active Raynaud attacks at inclusion, n (%)	18	10 (56)	14	4 (29)
Active digital ulcers at inclusion, n (%)	18	5 (28)	18	2 (11)
Telangiectasias, n (%)	18	16 (89)	18	16 (89)
Calcinosis cutis, n (%)	18	4 (22)	15	6 (40)
**Biological data**				
ESR (mm/h), mean ± SD	17	16 ± 8.6	16	19 ± 13
CRP (mg/L), mean ± SD	18	3.8 ± 1.8	18	6.6 ± 5.2
Creatinin (mg/L), mean ± SD	18	7.1 ± 1.6	18	8.7 ± 1.8
Estimated GFR (mL/min/1,73m^2^), mean ± SD	18	92 ± 29	18	73 ± 20
Nt-pro-BNP (pg/mL), mean ± SD	18	150 ± 123	18	950 ± 1241
Ferritin (ng/mL), mean ± SD	18	81 ± 73	18	58 ± 44
Uric acid (mg/L), mean ± SD	18	46 ± 11	17	61 ± 24
Complement activation, n (%)	18	0 (0)	17	0 (0)
**Transthoracic echocardiography**				
Left ventricular ejection fraction (%), mean ± SD	16	64 ± 8.2	16	66 ± 6.6
Left ventricular diastolic dysfunction, n (%)	16	4 (25)	17	7 (41)
Peak TRV (m/s), mean ± SD	14	2.46 ± 0.37	16	3.63 ± 0.65
RA area (cm^2^), mean ± SD	17	14 ± 4.8	18	21 ± 7.7
**Pulmonary function tests**				
TLC (% predicted), mean ± SD	17	104 ± 12	16	92 ± 9.3
FVC (% predicted), mean ± SD	18	117 ± 16	18	102 ± 17
DLCO (% predicted), mean ± SD	18	70 ± 13	18	38 ± 11
KCO (% predicted), mean ± SD	18	76 ± 13	18	45 ± 11
FVC (% predicted)/DLCO (% predicted) ratio, mean ± SD	18	1.73 ± 0.35	18	2.86 ± 0.76
**Right heart catheterization**				
At PAH diagnosis				
Time between RHC and inclusion (months), mean ± SD			18	34 ± 26
RAP (mmHg), mean ± SD			18	5.7 ± 3.2
sPAP (mmHg), mean ± SD			18	60 ± 16
dPAP (mmHg), mean ± SD			18	21 ± 6.5
mPAP (mmHg), mean ± SD			18	36 ± 10
PAWP (mmHg), mean ± SD			18	7.9 ± 2.3
Cardiac output (L/min), mean ± SD			18	4.7 ± 1.0
Cardiac index (L/min/m²), mean ± SD			18	2.8 ± 0.6
SvO_2_ (%), mean ± SD			16	69 ± 6
PVR (Wood units), mean ± SD			18	6.13 ± 2.38
Latest available RHC data				
Time between RHC and inclusion (months), mean ± SD			18	12 ± 14
RAP (mmHg), mean ± SD			17	5.5 ± 3.4
sPAP (mmHg), mean ± SD			17	57 ± 16
dPAP (mmHg), mean ± SD			17	26 ± 24
mPAP (mmHg), mean ± SD			18	35 ± 9.3
PAWP (mmHg), mean ± SD			18	9.1 ± 3.2
Cardiac output (L/min), mean ± SD			18	5.1 ± 1.3
Cardiac index (L/min/m²), mean ± SD			18	3.0 ± 0.6
SvO_2_ (%), mean ± SD			16	66 ± 9.6
PVR (Wood units), mean ± SD			18	5.6 ± 2.9
**Composite scores**				
EScSG-AI score, mean ± SD	18	1.17 ± 1.32	18	1.44 ± 1.21
Medsger severity score, mean ± SD	18	0.78 ± 1.48	16	1.26 ± 1.93
sHAQ score, mean ± SD	13	0.87 ± 0.67	15	1.01 ± 0.54
**Treatments at inclusion**				
Corticosteroids, n (%)	18	0 (0)	18	0 (0)
Immunosuppressants, n (%)	18	0 (0)	18	0 (0)
Hydroxychloroquine, n (%)	18	0 (0)	18	0 (0)
PAH specific drugs*, n (%)	18	1 (6)	18	12 (67)
Phosphodiesterase 5 inhibitors, n (%)		1 (6)		12 (67)
Endothelin receptor antagonists, n (%)		0 (0)		8 (44)
Prostacyclin analogues, n (%)		0 (0)		1 (6)
Oxygen, n (%)	18	0 (0)	18	4 (22)

ANA, antinuclear antibodies; BMI, body mass index; CRP, C-reactive protein; dc, diffuse cutaneous; DLCO, diffusing capacity of the lung for carbon monoxide; dPAP, diastolic pulmonary arterial pressure; EScSG-AI, European Scleroderma Study Group Activity Index; ESR, erythrocyte sedimentation rate; FVC, forced vital capacity; GFR, glomerular filtration rate; sHAQ, scleroderma Health Assessment Questionnaire; ILD, interstitial lung disease; KCO, diffusing coefficient for carbon monoxide; lc, limited cutaneous; mPAP, mean systolic pulmonary arterial pressure; Nt-pro-BNP, N-terminal prohormone of brain natriuretic peptide; NYHA, New York Heart Association; PAH, pulmonary arterial hypertension; PAWP, pulmonary arterial wedge pressure; PVR, pulmonary vascular resistance; RA, right atrium; RAP, right atrial pressure; sPAP, systolic pulmonary arterial pressure; SSc, systemic sclerosis; SvO_2_, venous saturation in oxygen; TLC, total lung capacity; TRV, tricuspid regurgitation velocity.

*In SSc-no PAH patients, these drugs were prescribed for digital ulcers and/or refractory Raynaud phenomenon.

**Figure 3:**
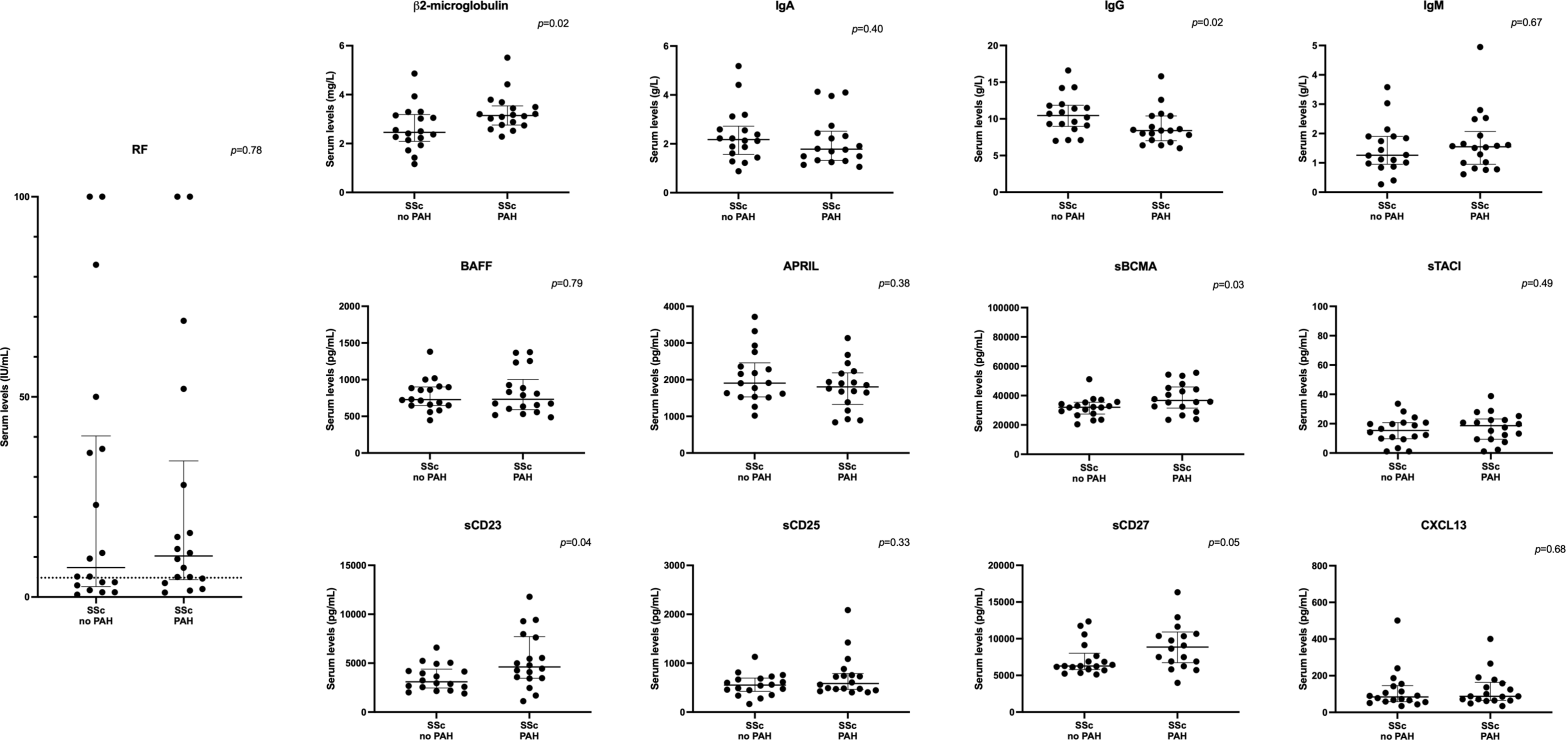
Serum levels of soluble markers of B cell activation in SSc patients with and without PAH (validation cohort). APRIL, a proliferation-inducing ligand; BAFF, B-cell-activating factor; BCMA, B-cell maturation antigen; CD, cluster of differentiation; CXCL13, C-X-C motif chemokine 13; HC, healthy controls; Ig, immunoglobulin; PAH, pulmonary arterial hypertension; RF, rheumatoid factor; s, soluble; SSc, systemic sclerosis; TACI, transmembrane activator and CAML interactor. The dotted line on the RF panel represents the threshold for RF positivity. Error bars display means and standard deviations.

In SSc patients with PAH, we observed significantly higher median levels of β2-microglobulin (3.15 [2.76;3.49] *vs.* 2.46 [2.14;3.15] mg/L, *p*=0.02), sBCMA (36699 [32360;45190] *vs.* 32,068 [27656;35352] pg/mL, *p*=0.03), sCD23 (4609 [3469;7628] *vs.* 3094 [2554;4200] pg/mL, *p*=0.04), and sCD27 (8859 [6889;10667] *vs.* 6282 [5825;7645], *p*=0.05), and lower median levels of IgG (8.40 [7.10;10.40] *vs.* 10.45 [9.10;11.80] g/L, *p*=0.02), compared to those without PAH (**Table 5**; **Figure 3**). There was no difference in serum levels of RF, IgA, IgM, BAFF, APRIL, sTACI, sCD25 and CXCL13 between the 2 groups.

**Table 5:** Serum levels of soluble markers of B cell activation in SSc patients with and without PAH (validation cohort).

Biomarkers	SSc-no PAH(N=18)	SSc-PAH(N=18)	Effect size	*p*-values
**RF** (IU/mL), median (Q1;Q3)	7.35 (2.90; 37.00)	10.25 (4.60; 28.00)	0.1 (-0.55; 0.75)	0.78
**β2-microglobulin** (mg/L), median (Q1;Q3)	2.46 (2.14; 3.15)	3.15 (2.76; 3.49)	0.89 (0.2; 1.57)	**0.02**
**IgA** (g/L), median (Q1;Q3)	2.18 (1.61; 2.59)	1.78 (1.33; 2.44)	-0.28 (-0.94; 0.37)	0.40
**IgG** (g/L), median (Q1;Q3)	10.45 (9.10; 11.80)	8.40 (7.10; 10.40)	-0.85 (-1.53; -0.17)	**0.02**
**IgM** (g/L), median (Q1;Q3)	1.26 (0.98; 1.90)	1.55 (1.00; 1.93)	0.15 (-0.51; 0.8)	0.67
**BAFF** (pg/mL), median (Q1;Q3)	727.5 (649.1; 897.9)	731.5 (602.3; 925.3)	-0.09 (-0.75; 0.56)	0.79
**APRIL** (pg/mL), median (Q1;Q3)	1907 (1532; 2358)	1804 (1384; 2169)	-0.29 (-0.95; 0.36)	0.38
**sBCMA** (pg/mL), median (Q1;Q3)	32068 (27656; 35352)	36699 (32360; 45190)	0.76 (0.08; 1.44)	**0.03**
**sTACI** (pg/mL), median (Q1;Q3)	15.35 (9.88; 20.67)	18.61 (9.39; 22.49)	0.24 (-0.42; 0.89)	0.49
**sCD23** (pg/mL), median (Q1;Q3)	3094 (2554; 4200)	4609 (3469; 7628)	0.72 (0.05; 1.39)	**0.04**
**sCD25** (pg/mL), median (Q1;Q3)	552.6 (448.2; 686.0)	584.2 (471.6; 756.6)	0.33 (-0.33; 0.98)	0.33
**sCD27** (pg/mL), median (Q1;Q3)	6282 (5825; 7645)	8859 (6889; 10667)	0.71 (0.03; 1.38)	**0.05**
**CXCL13** (pg/mL), median (Q1;Q3)	83.82 (59.15; 142.3)	87.49 (64.77; 158.4)	0.14 (-0.51; 0.79)	0.68

APRIL, a proliferation-inducing ligand; BAFF, B-cell-activating factor; BCMA, B-cell maturation antigen; CD, cluster of differentiation; CXCL13, C-X-C motif chemokine 13; FDR, false discovery rate; Ig, immunoglobulin; IU, international units; PAH, pulmonary hypertension; Q, quartile; RF, rheumatoid factor; s, soluble; SSc, systemic sclerosis; TACI, transmembrane activator and CAML interactor.

Results are expressed as median (first quartile; third quartile).

Effect sizes were calculated using the Cohen d. Absolute values of 0.20–0.49 represent a small change; values of 0.50–0.79 a medium change; and values of ≥ 0.80 a large change. Groups were matched on age (± 5 years) and gender. Given the exploratory nature of this analysis, p-values were not corrected for multiplicity.

**Figure 4:**
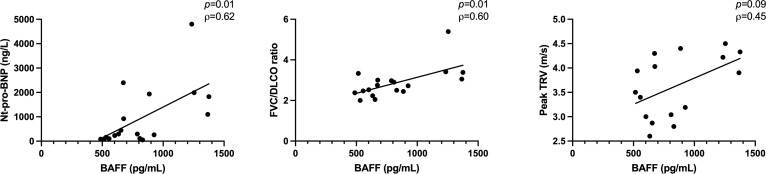
Associations between BAFF and clinical markers of PH, assessed among the 18 SSc-PAH patients from the validation cohort. BAFF, B-cell-activating factor; DLCO, diffusing capacity of the lung for carbon monoxide; FVC, forced vital capacity; Nt-pro-BNP, N-terminal prohormone of brain natriuretic peptide; PAH, pulmonary arterial hypertension; SSc, systemic sclerosis; TRV, tricuspid regurgitation velocity. Error bars display means and standard deviations. *p*-values are adjusted for age.

Next, we assessed the associations between serum levels of soluble markers of B cell activation and clinical markers of PH in the SSc-PAH group (**Table 6**). We identified moderate to strong associations of BAFF with Nt-pro-BNP levels (*p*=0.01, ρ=0.62), FVC/DLCO ratio (ρ=0.60, *p*=0.01) and peak TRV (ρ=0.45, *p*=0.09) (**Figure 4**). Peak TRV also correlated moderately with IgM (ρ=-0.59, *p*=0.02); and FVC/DLCO ratio with β2-microglobulin (ρ=0.43, *p*=0.08) and IgG (ρ=-0.41, *p*=0.10).

**Table 6:** Associations between serum levels of soluble markers of B cell activation and clinical markers of PH assessed among the 18 SSc-PAH patients from the validation cohort.

	RF(IU/mL)	β2m(mg/L)	IgA(g/L)	IgG(g/L)	IgM(g/L)	BAFF(pg/mL)	APRIL(pg/mL)	sBCMA(pg/mL)	sTACI(pg/mL)	sCD23(pg/mL)	sCD25(pg/mL)	sCD27(pg/mL)	CXCL13(pg/mL)
**Nt-pro-BNP** (pg/mL)	p=0.18 ρ=0.34 (-0.18;0.7)	p=0.37 ρ=0.23 (-0.29;0.64)	p=0.35 ρ=-0.24 (-0.64;0.28)	p=0.34 ρ=-0.25 (-0.65;0.27)	p=0.50 ρ=-0.18 (-0.60;0.34)	**p=0.01** ρ=0.62 (0.17;0.84)	p=0.10 ρ=0.41 (-0.10;0.74)	p=0.26 ρ=0.29 (-0.23;0.67)	p=0.56 ρ=0.15 (-0.36;0.59)	p=0.62 ρ=0.13 (-0.38;0.57)	p=0.24 ρ=0.30 (-0.22;0.68)	p=0.82 ρ=0.06 (-0.43;0.52)	p=0.57 ρ=0.15 (-0.36;0.58)
**Peak TRV** (m/s)	p=0.79 ρ=0.08 (-0.46;0.56)	p=0.34 ρ=0.27 (-0.29;0.68)	p=0.19 ρ=-0.36 (-0.73;0.2)	p=0.24 ρ=-0.32 (-0.71;0.24)	**p=0.02** ρ=-0.59 (-0.84;-0.09)	p=0.09 ρ=0.45 (-0.1;0.77)	p=0.06 ρ=0.50 (-0.04;0.80)	p=0.18 ρ=0.37 (-0.19;0.73)	p=0.06 ρ=0.49 (-0.05;0.79)	p=0.69 ρ=0.11 (-0.43;0.59)	p=0.47 ρ=0.20 (-0.35;0.64)	p=0.77 ρ=-0.08 (-0.57;0.45)	p=0.44 ρ=0.21 (-0.34;0.65)
**FVC/DLCO** ratio	p=0.65 ρ=-0.12 (-0.56;0.39)	p=0.08 ρ=0.43 (-0.07;0.75)	p=0.68 ρ=0.11 (-0.40;0.56)	p=0.10 ρ=-0.41 (-0.74;0.10)	p=0.53 ρ=-0.16 (-0.59;0.35)	**p=0.01** ρ=0.60 (0.15;0.83)	p=0.73 ρ=0.09 (-0.41;0.54)	p=0.95 ρ=0.02 (-0.47;0.49)	p=0.28 ρ=0.28 (-0.24;0.66)	p=0.83 ρ=0.06 (-0.44;0.52)	p=0.45 ρ=0.20 (-0.32;0.62)	p=0.93 ρ=-0.02 (-0.50;0.46)	p=0.99 ρ=0.004 (-0.48;0.48)

APRIL, a proliferation-inducing ligand; BAFF, B-cell-activating factor; BCMA, B-cell maturation antigen; CD, cluster of differentiation; CXCL13, C-X-C motif chemokine 13; DLCO, diffusing capacity of the lung for carbon monoxide; FVC, forced vital capacity; Ig, immunoglobulin; IU, international unit; Nt-pro-BNP, N-terminal prohormone of brain natriuretic peptide; PH, pulmonary hypertension; PAH, pulmonary arterial hypertension; RF, rheumatoid factor; s, soluble; SSc, systemic sclerosis; TACI, transmembrane activator and CAML interactor; TRV, tricuspid regurgitation velocity; β2m, β2-microglobulin.

All analyses are adjusted for age. Given the exploratory nature of this analysis, p-values were not corrected for multiplicity.

Effect sizes are expressed as the partial Spearman correlation coefficient ρ (with 95% confidence interval). The usual interpretation is that an absolute value of correlation between 0.00 to 0.19 is very weak; 0.20 to 0.39 weak; 0.40 to 0.59 moderate; 0.60 to 0.79 strong; and greater than 0.80 very strong.

Overall, these data suggest that soluble markers of B cell activation are differentially expressed in SSc-PAH patients and correlate with clinical markers of PH, especially for BAFF.

### B cells from SSc patients showed differential production of various pro- and anti-angiogenic mediators compared to HC

As our previous results suggested a link between B cell activation and SSc-PAH, we next wondered if B cells could be involved in the pathogenesis of SSc microangiopathy. In order to investigate this hypothesis, we tried to determine whether SSc B cells can produce proteins involved in angiogenic processes. As pathogenic antibodies against endothelial antigens have already been described in SSc patients (69–80), we chose to focus on non-Ig angiogenic mediators.

**Figure 5:**
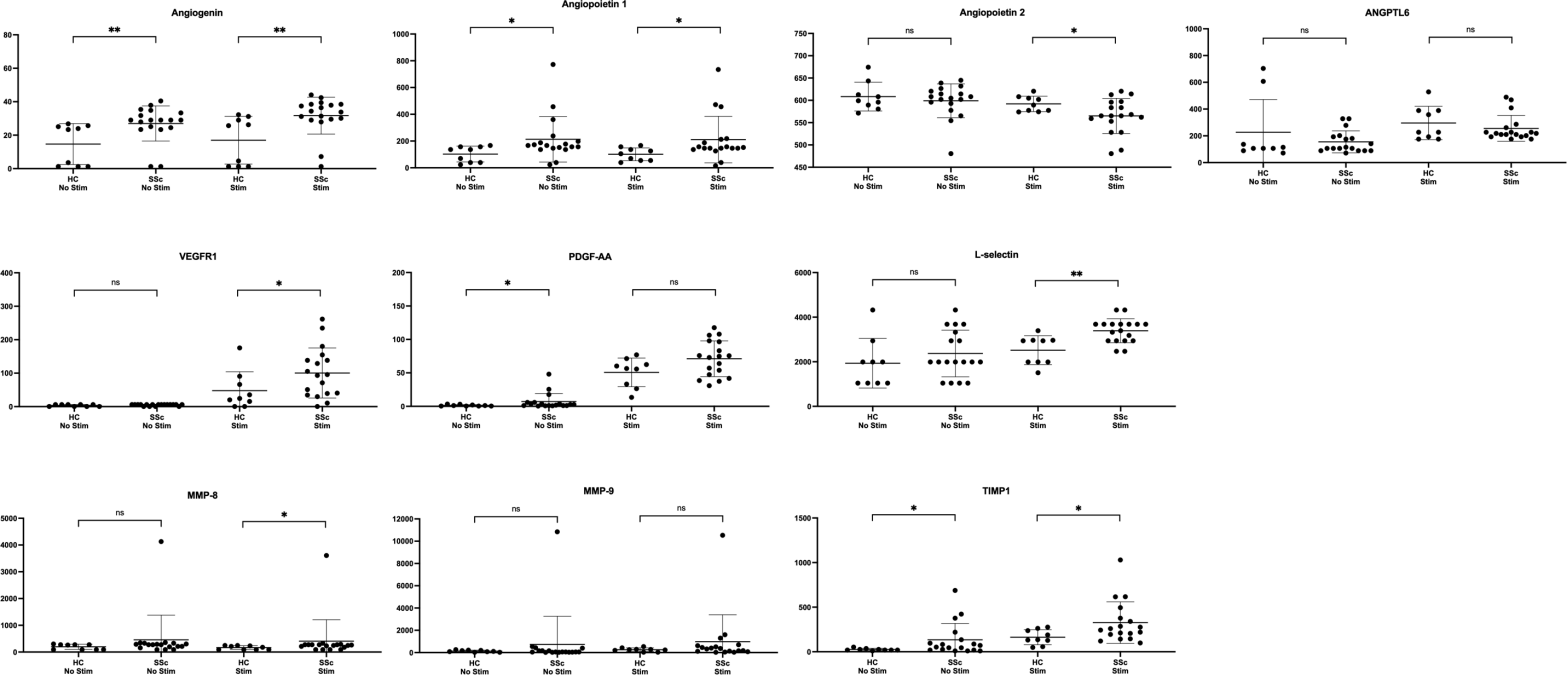
Concentrations of angiogenic mediators in B cell culture supernatants from SSc patients and healthy controls. ANGPTL-6, angiopoietin-like protein 6; HC, healthy control; MMP, matrix metallopeptidase; PDGF-AA, platelet derived growth factor AA; SSc, systemic sclerosis; stim, stimulation; TIMP-1, tissue inhibitor of metalloproteinases; VEGFR-1, vascular endothelial growth factor receptor 1. Results are pooled from 2 independent experiments. Error bars display means and standard deviations. Levels of significance are pictured as follows: ** for *p ≤* 0.01, * for *p ≤* 0.05, ns for *p* > 0.05. Given the exploratory nature of this analysis, *p*-values were not corrected for multiplicity. ns, not significant.

Circulating B cells from HC (n=9), and SSc patients with (n=9) and without (n=9) PAH from our validation cohort were cultured for 48h with and without stimulation. Our *in vitro* stimulation of B cells was designed to mimic an antigen- and T cell-dependent stimulation in presence of Toll-like receptor ligand and BAFF, as it is believed to occur in SSc patients. Groups were matched on age (± 5 years) and gender. Concentrations of several angiogenic mediators were assessed in culture supernatants (**Table 7**; **Figure 5**).

**Table 7:** Concentrations of angiogenic mediators in B cell culture supernatants from healthy controls, and SSc patients with and without PAH from the validation cohort.

		Healthy controls vs SSc patients				SSc-no PAH vs SSc-PAH patients			
		HC (N=9)	SSc (N=18)	Effect size	p	SSc-no PH(N=9)	SSc-PAH(N=9)	Effect size	p
**Angiogenin** (pg/mL) median (Q1;Q3)	No stim	23.35 (1.28; 25.09)	29.01 (24.51; 33.30)	1.36 (0.48; 2.24)	**0.008**	29.01 (23.40; 31.71)	29.01 (25.09; 34.86)	0.21 (-0.72; 1.13)	0.69
	Stim	25.66 (1.28; 29.01)	34.08 (29.55; 38.44)	1.49 (0.60; 2.38)	**0.004**	31.40 (30.10; 36.41)	37.43 (29.55; 38.44)	0.29 (-0.64; 1.21)	0.57
**Angiopoietin 1** (pg/mL) median (Q1;Q3)	No stim	136.3 (39.20; 156.9)	167.1 (146.6; 197.7)	1.15 (0.29; 2.00)	**0.02**	172.3 (167.1; 197.7)	156.9 (146.6; 187.6)	-0.33 (-1.26; 0.60)	0.51
	Stim	105.2 (54.68; 136.3)	149.2 (146.6; 212.9)	1.00 (0.16; 1.84)	**0.03**	146.6 (146.6; 212.9)	156.9 (136.3; 187.6)	-0.06 (-0.99; 0.86)	0.93
**Angiopoietin 2** (pg/mL) median (Q1;Q3)	No stim	599.0 (589.4; 612.6)	606.5 (592.9; 620.4)	0.05 (-0.75; 0.85)	0.92	608.2 (592.9; 620.4)	604.9 (595.9; 614.3)	-0.08 (-1.01; 0.84)	0.89
	Stim	589.4 (577.5; 604.9)	565.2 (552.9; 595.9)	-0.92 (-1.76; -0.08)	**0.05**	565.2 (559.1; 612.6)	565.2 (552.9; 583.6)	-0.37 (-1.30; 0.56)	0.45
**ANGPTL-6** (pg/mL) median (Q1;Q3)	No stim	106.5 (106.5; 136.4)	110.8 (89.03; 192.8)	0.03 (-0.77; 0.83)	0.96	106.5 (89.03; 175.6)	115.2 (106.5; 192.8)	0.18 (-0.74; 1.11)	0.72
	Stim	227.1 (192.8; 388.9)	218.6 (201.4; 261.3)	-0.2 (-1.00; 0.6)	0.62	227.1 (201.4; 227.1)	210.0 (210.0; 261.3)	-0.08 (-1.01; 0.84)	0.89
**VEGFR-1** (pg/mL) median (Q1;Q3)	No stim	6.16 (0.35; 6.16)	6.16 (6.16; 6.16)	0.46 (-0.35; 1.27)	0.26	6.16 (6.16; 6.16)	6.16 (6.16; 6.16)	0.00 (-0.92; 0.92)	1.00
	Stim	24.29 (15.40; 66.24)	94.49 (39.36; 138.4)	0.90 (0.07; 1.74)	**0.04**	93.28 (39.36; 138.4)	95.70 (51.02; 138.4)	0.08 (-0.84; 1.01)	0.89
**PDGF-AA** (pg/mL) median (Q1;Q3)	No stim	1.21 (0.42; 1.99)	2.71 (1.21; 5.87)	0.95 (0.11; 1.79)	**0.04**	2.71 (1.22; 3.78)	1.79 (1.21; 5.87)	-0.10 (-1.03; 0.82)	0.86
	Stim	56.54 (33.29; 62.46)	73.97 (47.33; 96.53)	0.77 (-0.06; 1.59)	0.09	74.88 (63.92; 97.90)	57.15 (47.33; 83.17)	-0.31 (-1.24; 0.62)	0.54
**L-selectin** (pg/mL) median (Q1;Q3)	No stim	1988 (1042; 1988)	1988 (1988; 3329)	0.46 (-0.35; 1.27)	0.27	1988 (1988; 3329)	1988 (1988; 2939)	-0.17 (-1.09; 0.76)	0.75
	Stim	2939 (1988; 2961)	3539 (2939; 3684)	1.34 (0.47; 2.22)	**0.007**	3329 (2939; 3684)	3684 (3184; 3684)	0.19 (-0.74; 1.12)	0.71
**MMP-8** (pg/mL) median (Q1;Q3)	No stim	260.4 (92.43; 274.9)	274.9 (195.7; 328.4)	0.68 (-0.14; 1.50)	0.12	274.9 (274.9; 328.4)	213.1 (195.7; 288.9)	-0.27 (-1.19; 0.66)	0.59
	Stim	177.2 (92.43; 229.6)	252.9 (177.2; 274.9)	1.00 (0.16; 1.84)	**0.04**	260.4 (245.3; 274.9)	229.6 (177.2; 274.9)	-0.06 (-0.99; 0.86)	0.93
**MMP-9** (pg/mL) median (Q1;Q3)	No stim	111.4 (77.59; 198.1)	56.22 (39.83; 164.7)	-0.30 (-1.11; 0.50)	0.49	53.64 (39.83; 164.7)	58.80 (46.75; 161.7)	0.16 (-0.76; 1.09)	0.76
	Stim	260.7 (198.1; 293.9)	345.4 (118.2; 618.1)	0.30 (-0.51; 1.10)	0.50	406.5 (323.4; 513.5)	163.4 (118.2; 618.1)	-0.14 (-1.07; 0.78)	0.79
**TIMP-1** (pg/mL) median (Q1;Q3)	No stim	19.28 (19.28; 26.57)	62.09 (19.28; 135.4)	0.94 (0.10; 1.78)	**0.05**	52.86 (26.57; 95.98)	81.82 (19.28; 135.4)	0.02 (-0.90; 0.94)	1.00
	Stim	135.4 (128.2; 237.5)	251.1 (194.9; 376.7)	0.99 (0.14; 1.83)	**0.03**	219.4 (206.0; 339.9)	259.6 (145.2; 376.7)	0.02 (-0.90; 0.94)	1.00

ANGPTL-6, angiopoietin-like protein 6; HC, healthy control; MMP, matrix metallopeptidase; PAH, pulmonary arterial hypertension; PDGF-AA, platelet derived growth factor AA; SSc, systemic sclerosis; stim, stimulation; TIMP-1, tissue inhibitor of metalloproteinases; VEGFR-1, vascular endothelial growth factor receptor 1.

Results are pooled from 2 independent experiments and expressed as median (first quartile; third quartile).

Effect sizes were calculated using the Cohen d. Absolute values of 0.20–0.49 represent a small change; values of 0.50–0.79 a medium change; and values of ≥ 0.80 a large change. Given the exploratory nature of this analysis, p-values were not corrected for multiplicity.

We observed higher production of angiogenin (*p*=0.004 and *p*=0.008), angiopoietin 1 (*p*=0.03 and *p*=0.02), PDGF-AA (*p*=0.09 and *p*=0.04) and TIMP-1 (*p*=0.03 and *p*=0.05) in B cell culture supernatants from SSc patients compared to HC, both with and without stimulation (respectively). We also found lower levels of angiopoietin 2 (*p*=0.05) as well as higher levels of VEGFR-1 (*p*=0.04) and MMP-8 (*p*=0.04) in supernatants of SSc B cells only after stimulation. Concentrations of ANGPTL-6 and MMP-9 were similar in supernatants from SSc patients and HC, both with and without stimulation. There was no detectable or negligeable B cell production of any of the other selected angiogenic mediators in any groups and regardless of stimulation.

When comparing supernatant levels of SSc patients with and without PAH, we observed no significant difference in B cell secretion of any of these angiogenic factors, in both stimulation conditions.

Overall, these results suggest that B cell produce angiogenic mediators in SSc patients, although with no significant difference in case of PAH.

## Discussion

To our knowledge, this is the first study that assessed a wide panel of soluble markers of B cell activation in 2 large and well-characterized SSc patient cohorts. Our results can be summarized as follows: 1/in a discovery cohort of SSc patients, we identified associations of B cell biomarkers with SSc phenotype, disease activity and severity, ILD and PH; 2/in this discovery population as well as in an independent validation cohort enriched with SSc-PAH patients, serum IgG, β2-microglobulin, BAFF, sBCMA and sCD23 levels were associated with PH status and/or clinical markers (**Table 8**); 3/B cells from SSc patients showed differential production of various pro- and anti-angiogenic mediators compared to HC.

**Table 8:** Synthetic view of the associations identified between soluble markers of B cell activation and clinical markers of PH in the discovery and validation cohorts.

Discovery cohort		Validation cohort	
PH *vs* no PH	**IgG** **RF**	PAH *vs* no PAH	**IgG** **β2-microglobulin** **sBCMA** **sCD23** **sCD27**
Nt-pro-BNP	**β2-microglobulin** sBCMA	Nt-pro-BNP	**BAFF**
Peak TRV	**BAFF** **RF**	Peak TRV	BAFF **IgM**
DLCO	**β2-microglobulin** BAFF sCD23 **RF**	FVC/DLCO	β2-microglobulin **BAFF** IgG

BAFF, B-cell-activating factor; BCMA, B-cell maturation antigen; CD, cluster of differentiation; DLCO, diffusing capacity of the lung for carbon monoxide; Nt-pro-BNP, N-terminal prohormone of brain natriuretic peptide; PAH, pulmonary arterial hypertension; PH, pulmonary hypertension; RF, rheumatoid factor; s, soluble; TRV, tricuspid regurgitation velocity.

Bold characters denote statistical significance (p< 0.05). Regular characters denote trends for statistical significance (p< 0.10).

### Soluble markers of B cell activation in systemic sclerosis

Previous publications that studied B cell biomarkers in SSc reported various associations with disease characteristics, especially with cutaneous and interstitial lung involvements. RF positivity in SSc patients varied considerably between studies, ranging from 12 to 71%, with no clear relationship with articular involvement (39–45). In line with our results, circulating concentrations of β2-microglobulin were found increased in SSc patients, with no difference between cutaneous subsets, and correlated with erythrocyte sedimentation rate (30). Conversely, serum IgG levels were previously reported as similar in SSc patients and HC, although significant differences were found among IgG subclasses (38).

Associations of BAFF with SSc characteristics have been more thoroughly documented, yet with conflicting results that challenge comparisons with our work. Conversely to our study, previous publications have consistently observed elevated serum BAFF levels in SSc patients (10, 31, 33–36). Our result could be at least partly explained by a positive correlation between age and BAFF concentrations in our cohort; an unexpected result since BAFF levels have been described as negatively associated with age in HC (81) and SLE patients (82). Although we do not have a satisfying explanation for this phenomenon, it should be noted that these studies included patients with a very wide spectrum of ages; and it is not sure that this negative association observed over several decades of life remains true when focusing on shorter age ranges.

We did not observe any association of BAFF with skin involvement, ILD or autoantibody profile. Previous studies reported highly contradictory results: some observed significant associations with cutaneous subset and mRSS (35, 36) while others did not (31, 33, 34); serum BAFF levels were found positively (36), negatively (31) or not (33, 34) correlated with ILD; and association with autoantibody profile have been observed in one study (31) but not others (33, 34, 36). BAFF concentrations were also associated with CRP and EScSG-AI score (although not reaching statistical significance for the latter in our work), which was not observed in other publications (33, 35, 36). Overall, these discrepancies could be due to heterogeneities between study populations, especially since our discovery cohort was selected to include a wide range of SSc manifestations; and probably reflect a differential implication of B cells between patients, organ involvements, and disease stages.

Serum APRIL levels have been described as either similar (10) or increased (28, 29) compared to HC. Contrary to our work, no correlation was found with CRP levels or autoantibody profile; and associations with ILD were inconsistently observed (28, 29). CXCL13 levels were increased in SSc (10, 33, 37) and correlated with EScSG-AI score (an association that we also observed in our work, although not reaching statistical significance). Associations with cutaneous subset and ILD were inconsistently reported (33, 37).

Levels of sCD21 were found lower in SSc than in HC, especially in case of lcSSc and ILD, but not associated with autoantibody profile (46). Concentrations of sCD23 in SSc were higher than in HC in a single study (47). Previous publications regarding sCD25 levels in SSc found discordant results: sCD25 concentrations have been reported as both higher than (51, 52, 54–56) and similar to (49, 50) HC; and correlations with skin fibrosis (53, 54, 56) and ILD (48, 53, 54) were not consistently observed. However, associations with disease activity seemed more systematically reported (49, 50, 52, 56), a result that we also observed in our study.

We could not find previous reports of sCD27, sTACI and sBCMA levels in SSc patients.

Overall, these data suggest that soluble markers of B cell activation could be interesting tools to assess organ involvement, disease activity and severity in SSc patients. Future studies should try to further validate their relevance as diagnostic and prognostic biomarkers in this disease.

### B cell activation in SSc-PAH

A further original finding of our work is the identification of associations between several markers of B cell activation (IgG, β2-microglobulin, BAFF, sBCMA and sCD23) and PAH status and/or clinical markers in 2 independent populations. Few publications have focused on B cell biomarkers and PAH in SSc patients: serum BAFF, APRIL and CXCL13 levels were found similar between patients with and without PH (31, 33, 34, 37); but apart from one study that reported an almost-significant correlation between CXCL13 and TTE-estimated systolic pulmonary arterial pressure (37), associations with clinical markers of PH were not studied. Although some results overlap between our discovery and validation cohorts, B cell biomarkers associations with PH status and clinical markers were not exactly similar in the 2 populations (**Table 8**). This could be explained by differences in inclusion criteria between cohorts (and notably the presence of patients with group 2 and group 3 PH in the discovery cohort), but could also suggest heterogeneities in B cell activation among SSc patients with PH.

Aside from SSc, B cell biomarkers have also been studied in other causes of PH. For instance, serum concentrations of CXCL13 were increased in patients with idiopathic PAH, connective tissue diseases-associated PAH and chronic thromboembolic PH (83, 84) with weak correlations with hemodynamic parameters in this latter subgroup (84). In patients with Sjögren’s syndrome, occurrence of PAH was associated with higher RF titers and hypergammaglobulinemia (85, 86). Interestingly, a novel mutation of *TNFRSF13B*, the gene coding for TACI, was identified as responsible for a familial form of PAH in Japanese patients (87).

Several evidence pleads for a participation of B cell activation in the pathogenesis of SSc-PAH. Firstly, recent works have observed abnormal B cell homeostasis in SSc-PAH patients, with lower total B cell counts, expanded IgD^+^ naïve subset and decreased memory B cells (88). SSc-PAH B cells display features of activation, with membrane over-expression of CD25 and increased susceptibility to apoptosis (89); and B-cell related genes were amongst the most differentially expressed between SSc patients with and without PAH (90). Moreover, a recently described B cell population, characterized by a low membrane expression of CD21, appeared particularly associated with vascular events in SSc patients: indeed, the proportion of circulating CD21^low^ B cells was associated with sPAP, DLCO and FVC/DLCO ratio in SSc patients (91).

Secondly, there is also evidence of functional anomalies in SSc B cells that can contribute to pulmonary vasculopathy. Indeed, SSc-PAH B cells display anomalies in their antibody repertoire, with an over-representation of specific VDJ rearrangements and somatic hypermutations, suggesting small clonal selections and expansions (88). Furthermore, several autoantibodies have been identified in the serum of SSc patients, that seem to have a direct pathogenic effect on the endothelium: indeed, antibodies directed against endothelial cells, endothelin 1 receptor and angiotensin II receptor can induce activation and apoptosis of endothelial cells and are associated with PAH onset (69–80).

Finally, a recent randomized placebo-controlled trial tested the efficacy of rituximab to treat SSc-PAH patients (92). In the primary analysis based on longitudinal data through week 24, the adjusted mean change in the six-minute walk distance (6MWD) from baseline to 24 weeks did not differ significantly between arms. However, in a secondary analysis using 6MWD data through week 48, the rituximab arm was superior to placebo. Interestingly, machine learning analysis identified low levels of RF, as well as IL-12 and IL-17, as robust predictors to response to rituximab. The authors suggested that this phenomenon may be explained by a reduction of complement-dependent cytotoxicity due to the binding of RF to the Fc portion of rituximab.

Overall, these data further suggest the occurrence of B cell activation in SSc-PAH patients.

### B cells as producers of angiogenic mediators: A new player in SSc microangiopathy?

Another original finding of our study is the demonstration that SSc B cells can produce angiogenic factors, which to our knowledge has never been reported before in this disease. B cells have been shown to secrete various pro- and anti-angiogenic mediators (including angiopoietins, VEGFs and their receptors, PDGFs, MMPs and their inhibitors TIMPs) in other conditions, but essentially in the context of B cell neoplasias (93–102).

Angiogenesis is a highly regulated process that leads to the formation of newly formed capillaries from preexisting vessels (103). It is initiated by the release of proteolytic enzymes, such as matrix metalloproteinases (MMPs), resulting in the degradation of the endothelial cell basement membrane and the perivascular extracellular matrix (103). Following matrix degradation, endothelial cells activate, proliferate and migrate into the surrounding area, forming vessel sprouts, under the effect of specific proangiogenic factors such as angiogenin, angiopoietin 1, VEGFs and PDGFs (103). Proteins that inhibit matrix degradation (like TIMPs) or endothelial cell mobilization (such as angiopoietin 2, a natural antagonist of angiopoietin 1) thus have angiostatic properties (103). During SSc, chronic vascular injury induces hypoxia and tissue ischemia, which are classical triggers of angiogenesis, but eventually leads to loss of capillaries and avascular areas: as such, angiogenesis is generally considered to be dysregulated in SSc and insufficient to compensate for the loss of vasculature occurring during the disease (104, 105).

Dysregulation of several pro- and anti-angiogenic factors have been documented in SSc patients (106). Circulating angiogenin concentrations are elevated in SSc patients, with no correlation with Raynaud phenomenon or disease duration (107). SSc patients also displays an imbalance in angiopoietin production, with decreased serum levels of angiopoietin 1 and increased serum levels of angiopoietin 2 (associated with digital ulcers) (108). VEGF is strongly upregulated in the skin and serum of SSc patients, including its anti-angiogenic VEGF(165)b isoform. Studies on VEGFR-1 and -2 expression by skin endothelial cells have yielded conflicting results (109). PDGF levels are increased in the serum, skin and bronchoalveolar lavage of SSc patients (110); and stimulatory autoantibodies directed against PDGF receptors have also been documented (111). Dysregulated production of various MMPs and TIMPs was observed in the skin and serum of SSc patients (112, 113).

Although the balance between pro- and anti-angiogenic mediator dysregulation clearly favors the former, this does not seem sufficient to induce compensatory angiogenesis in SSc, which suggests that more complex mechanisms are at work to account for SSc vasculopathy (106). Uncontrolled chronic over-expression of proangiogenic mediators throughout various disease stages might contribute to disturbed vessel morphology and endothelial dysfunction rather than to promote new vessel formation (106). As such, it is difficult to predict how the production of angiogenic mediators by B cells would contribute to SSc microangiopathy. Further mechanistic works are warranted to elucidate the pathophysiological relevance of this finding.

### Strengths and limitations

Our study draws strength from its large and well-phenotyped study populations, the wide panel of biomarkers screened, the sample collection on the same day as clinical evaluation (which allowed to perform accurate correlations), the validation of our results on an independent cohort, and the confirmation of the pathophysiological relevance of our observations.

It also has limitations. Firstly, we included 2 selected SSc populations, which may limit the generalization of our findings. Secondly, as this was an exploratory, hypothesis-generating study, statistical analyses were not systematically corrected for multiplicity, which could have increased the α-risk score. Thirdly, most of the associations observed between B cell biomarkers and SSc characteristics have low to medium effect sizes, which could limit the scope of our findings. Fourthly, patients from the discovery cohort were treated by drugs targeting the immune system (corticosteroids, hydroxychloroquine, immunosuppressants), which could have influenced the serum levels of our B cell biomarkers, or biased the associations that were studied. However, we have tried to take their effect into account in our analyses by adjusting our associations on these treatments in our discovery population, and excluding patients treated with such drugs from our validation population. Fifthly, some patients in the discovery cohort had received rituximab infusions, although treatment was discontinued several years before inclusion for most of them. Finally, SSc-PAH patients were treated by PAH-specific drugs, which may have biased the associations between B cell biomarkers and PH clinical markers, and possibly the B cell production of angiogenic factors.

## Conclusion

Soluble markers of B cell activation could be relevant tools to assess organ involvements, disease activity and severity in SSc patients. Further studies with larger samples and longitudinal design are warranted to test their capacity to predict the occurrence of severe organ involvements. Notably, some of them showed interesting associations with PAH, which can plead for a role of B cell activation in the pathogenesis of the pulmonary microangiopathy. B cells could contribute to the SSc vasculopathy through the production of non-Ig angiogenic mediators.

## Data availability statement

The raw data supporting the conclusions of this article will be made available by the authors, without undue reservation.

## Ethics statement

The studies involving human participants were reviewed and approved by Comité de Protection des Personnes Sud-Est II. (CPP #2019-87, RCB and EUDRACT # 2019-A01083-54). The patients/participants provided their written informed consent to participate in this study.

## Author contributions

We confirm that all individuals listed as authors met all the required criteria for authorship. SS, TG, SD, and DL conceived and designed the study. SS and CP screened patients and collected patient data. GL contributed serum samples for the experiments. TG, LG, CH, AL, and MB performed the experiments. SS and CP constituted and managed the database. AD performed the statistical analyses. SS interpreted the results and wrote the first draft of the manuscript. TG, SD, and DL made major revisions to the manuscript. GL, MJ, and SSp provided their expertise on B cell biology. EH and VS provided their expertise on SSc and PAH. All authors read and approved the final submitted version of the manuscript and agreed to be accountable for all aspects of the work in ensuring that questions related to the accuracy or integrity of any part of the work are appropriately investigated and resolved.

## Conflict of interest

The authors declare that the research was conducted in the absence of any commercial or financial relationships that could be construed as a potential conflict of interest.

## Publisher’s note

All claims expressed in this article are solely those of the authors and do not necessarily represent those of their affiliated organizations, or those of the publisher, the editors and the reviewers. Any product that may be evaluated in this article, or claim that may be made by its manufacturer, is not guaranteed or endorsed by the publisher.
