# Supplementary material for: Soluble markers of B cell activation suggest a role of B cells in the pathogenesis of systemic sclerosis-associated pulmonary arterial hypertension
Source: Front Immunol. 2022 Jul 29;13:954007. doi: 10.3389/fimmu.2022.954007 (PMC9374103; doi:10.3389/fimmu.2022.954007)
Supplement: Supplementary file 1 [file DataSheet_1.pdf]

Supplemental Table 1. Conditions associated with RF positivity in the 44 RF-positive SSc patients from the discovery cohort.

	N	Value
**Immunologic diseases**		
Diffuse cutaneous SSc, n (%)	44	29 (66%)
Overlap syndrome, n (%)	43	19 (44%)
Sjogren's syndrome, n (%)	43	9 (21%)
Systemic *lupus erythematosus*, n (%)	43	4 (9%)
Inflammatory myopathy, n (%)	43	4 (9%)
Rheumatoid arthritis, n (%)	43	2 (5%)
**Infectious diseases**		
Hepatitis C, n (%)	19	0 (0%)
Hepatitis B, n (%)	20	0 (0%)
**Hematologic diseases**		
Lymphoma, n (%)	44	0 (0%)

RF: rheumatoid factor; SSc: systemic sclerosis.

Supplemental Table 2. Detailed characteristics of SSc patients treated by rituximab in the discovery cohort.

Patient	Age	SSc subset	ILD	PH	RTX regimen	RTX indication	Months since last RTX infusion
#1	62	lcSSc	extensive ILD	no	2 doses of 1g (D1 and D15)	progressive ILD	2
#2	27	dcSSc	extensive ILD	no	2 doses of 1g (D1 and D15)<br>followed by 4 doses of 500 mg<br>(1 dose every 6 months for 2 years)	progressive ILD	3
#3	55	lcSSc	extensive ILD	no	2 doses of 1g (D1 and D15)<br>followed by 1 dose of 500 mg	progressive ILD	9
#4	46	lcSSc	extensive ILD	no	2 doses of 1g (D1 and D15)	joint involvement	13
#5	83	dcSSc	limited ILD	no	2 doses of 1g (D1 and D15)	progressive skin fibrosis	32
#6	53	dcSSc	extensive ILD	no	2 doses of 1g (D1 and D15)<br>followed by 1 dose of 500 mg	progressive ILD	44
#7	60	dcSSc	extensive ILD	no	2 doses of 1g (D1 and D15)<br>followed by 1 dose of 500 mg	progressive ILD	83

D: day; dc: diffuse cutaneous; ILD: interstitial lung disease; lc: limited cutaneous; PH: pulmonary hypertension; RTX: rituximab; SSc: systemic sclerosis.

Supplemental Table 3. Serum levels of soluble markers of B cell activation in the SSc patients and healthy controls (discovery cohort), after exclusion of the 7 SSc patients treated by rituximab.

**Biomarkers**	**Healthy controls** (N=80)	**SSc patients never treated by RTX** (N=73)	**Effect size**[1]	***p*-values**[2]	***p*-values adjusted for FDR**[3]
**Positive RF**, n (%)	8 (10%)	40 (55%)	12.1 (4.4 ; 33.1)	<0.0001	**<0.0001**
**β2-microglobulin** (mg/L), median (Q1;Q3)	1.55 (1.34 ; 1.74)	2.11 (1.80 ; 2.65)	0.64 (0.37 ; 0.91)	<0.0001	**<0.0001**
**IgA** (g/L), median (Q1;Q3)	1.82 (1.31 ; 2.45)	2.10 (1.67 ; 2.93)	0.41 (0.09 ; 0.72)	0.01	**0.04**
**IgG** (g/L), median (Q1;Q3)	9.30 (7.96 ; 10.31)	9.53 (8.34 ; 12.10)	0.62 (0.31 ; 0.94)	0.0001	**0.0004**
**IgM** (g/L), median (Q1;Q3)	0.81 (0.61 ; 1.20)	1.01 (0.62 ; 1.55)	0.11 (-0.19 ; 0.40)	0.48	0.55
**BAFF** (pg/ml), median (Q1;Q3)	534 (446 ; 624)	589 (453 ; 812)	-0.18 (-0.49 ; 0.12)	0.25	0.38
**APRIL** (pg/ml), median (Q1;Q3)	1911 (1619 ; 2236)	1948 (1408 ; 2297)	-0.14 (-0.46 ; 0.18)	0.39	0.53
**sBCMA** (pg/ml), median (Q1;Q3)	37344 (29070 ; 47014)	44570 (26488 ; 58982)	0.37 (0.05 ; 0.69)	0.02	0.05
**sTACI** (pg/ml), median (Q1;Q3)	3.79 (1.64 ; 7.18)	5.27 (2.14 ; 11.80)	0.28 (-0.04 ; 0.60)	0.94	0.94
**sCD21** (pg/ml), median (Q1;Q3)	51516 (39990 ; 64006)	47551 (33477 ; 58844)	-0.13 (-0.44 ; 0.18)	0.41	0.53
**sCD23** (pg/ml), median (Q1;Q3)	1952 (1405 ; 3168)	1815 (1056 ; 3144)	-0.09 (-0.41 ; 0.22)	0.57	0.61
**sCD25** (pg/ml), median (Q1;Q3)	305 (246 ; 391)	321 (239 ; 564)	0.24 (-0.08 ; 0.56)	0.15	0.26
**sCD27** (pg/ml), median (Q1;Q3)	4440 (3615 ; 5658)	4919 (4221 ; 7463)	0.33 (0.02 ; 0.64)	0.04	0.08
**CXCL13** (pg/ml), median (Q1;Q3)	36.95 (24.46 ; 55.77)	81.69 (47.64 ; 120.0)	1.06 (0.72 ; 1.39)	<0.0001	**<0.0001**

APRIL: a proliferation-inducing ligand; BAFF: B-cell-activating factor; BCMA: B-cell maturation antigen; CD: cluster of differentiation; CXCL13: C-X-C motif chemokine 13; FDR: false discovery rate; Ig: immunoglobulin; Q: quartile; RF: rheumatoid factor; RTX: rituximab; s: soluble; SSc: systemic sclerosis; TACI: transmembrane activator and CAML interactor.
Results are expressed as median (first quartile; third quartile) for quantitative biomarkers and as frequency (percentage) otherwise.
All analyses were adjusted for age and gender.

[1] For quantitative biomarkers, effect sizes were calculated on log transformed variables using the Cohen *d*. Absolute values of 0.20–0.49 represent a small change; values of 0.50–0.79 a medium change; and values of ≥ 0.80 a large change. For the binary biomarker, effect size is the odds ratio of the status for the risk of positive RF with the status control as reference value.

[2] *p*-values calculated on log-transformed variables for quantitative biomarkers.

[3] *p*-values corrected for multiplicity using the False Discovery Rate (FDR) method (Benjamini Hochberg procedure).

Supplemental Figure 1. Assessment of B cell purity.

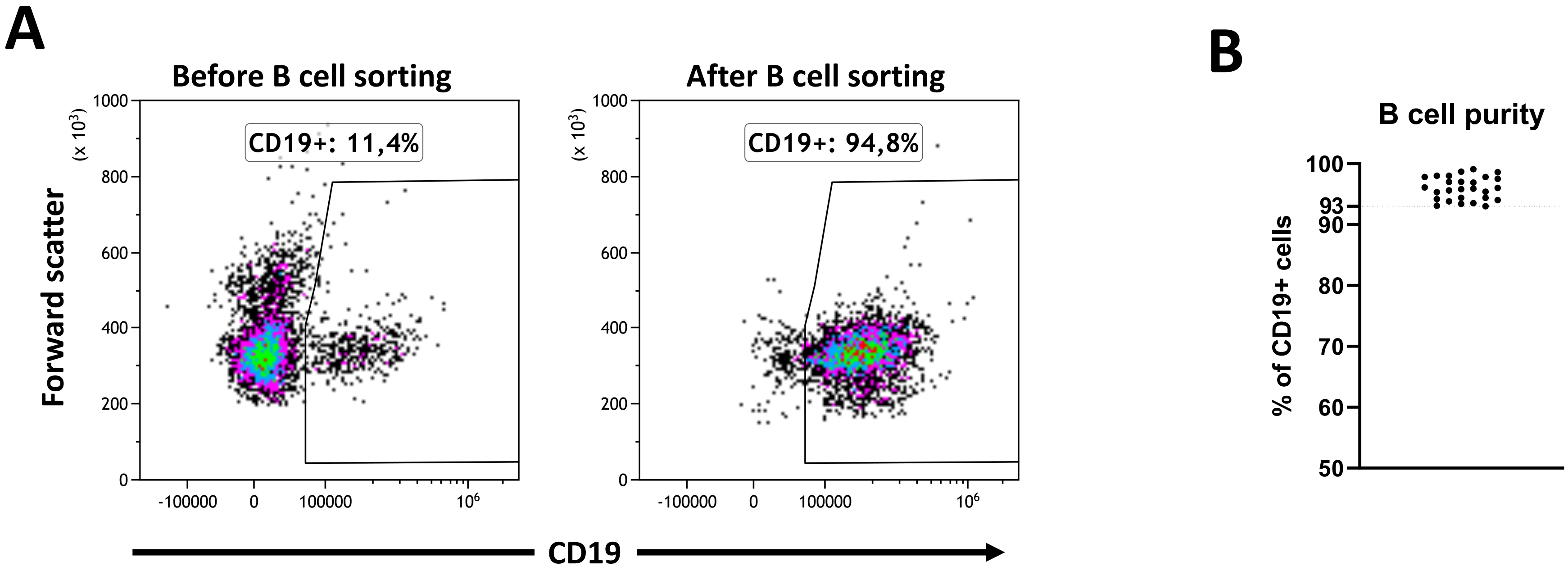

(A) For a representative sample of PBMCs, cytometry dot plots showing the proportion of CD19+ cells among PBMCs before B cell sorting and among the cell suspension obtained after B cell sorting. (B) Purity of sorted B cells assessed by cytometry as shown in (A).
